# The study of surface wetting, nanobubbles and boundary slip with an applied voltage: A review

**DOI:** 10.3762/bjnano.5.117

**Published:** 2014-07-15

**Authors:** Yunlu Pan, Bharat Bhushan, Xuezeng Zhao

**Affiliations:** 1 Mechanical Engineering, Harbin Institute of Technology, Harbin, 150001, P.R. China; 2 Nanoprobe Laboratory for Bio- & Nanotechnology and Biomimetics (NLB2), The Ohio State University, 201 W. 19th Avenue, Columbus, OH 43210-1142, USA

**Keywords:** atomic force microscopy, boundary slip, electrowetting, nanobubbles, surface charge

## Abstract

The drag of fluid flow at the solid–liquid interface in the micro/nanoscale is an important issue in micro/nanofluidic systems. Drag depends on the surface wetting, nanobubbles, surface charge and boundary slip. Some researchers have focused on the relationship between these interface properties. In this review, the influence of an applied voltage on the surface wettability, nanobubbles, surface charge density and slip length are discussed. The contact angle (CA) and contact angle hysteresis (CAH) of a droplet of deionized (DI) water on a hydrophobic polystyrene (PS) surface were measured with applied direct current (DC) and alternating current (AC) voltages. The nanobubbles in DI water and three kinds of saline solution on a PS surface were imaged when a voltage was applied. The influence of the surface charge density on the nanobubbles was analyzed. Then the slip length and the electrostatic force on the probe were measured on an octadecyltrichlorosilane (OTS) surface with applied voltage. The influence of the surface charge on the boundary slip and drag of fluid flow has been discussed. Finally, the influence of the applied voltage on the surface wetting, nanobubbles, surface charge, boundary slip and the drag of liquid flow are summarized. With a smaller surface charge density which could be achieved by applying a voltage on the surface, larger and fewer nanobubbles, a larger slip length and a smaller drag of liquid flow could be found.

## Introduction

The interface of solid and liquid plays an important role in liquid flow in various fluidics based micro/nano-electro-mechanical systems (MEMS/NEMS), which have a large surface area to volume ratio [1–2]. At the interface of solid and liquid, surface wetting, surface charge, nanobubbles and boundary slip are believed to affect the drag of liquid flow [3–10]. By applying a voltage to the system, the surface wettability can be changed, known as electrowetting, and the surface charge density can be changed as well [11]. Nanobubbles and boundary slip are believed to have a strong influence on surface wettability and surface charge [12–17]. It can be inferred that when a voltage is applied, the surface wettability, surface charge, nanobubbles and boundary slip will be changed, which causes the change in the drag of liquid flow. The study of the influence of the applied voltage on the surface wettability, surface charge, nanobubbles and boundary slip is important and necessary for the minimization of the drag of liquid flow.

On some hydrophobic surfaces, the fluid velocity near the solid surface is not zero on the solid surface [18–22]. This phenomenon, the so called boundary slip condition, affects the boundary condition of Navier–Stokes equation which is widely used to describe the liquid flow, and is represented by the slip length. The boundary slip condition has been studied by various groups [10,12,15,23–26]. Most studies have a similar goal that is to find a method to affect the boundary slip condition by increasing the boundary slip length, which then reduces the drag of fluid flow. Recently, it is found that a higher hydrophobicity can provide a larger slip length [17]. Very large slip lengths were found on superhydrophobic surfaces [6,27–31]. However, more convenient methods that can increase the boundary slip length without changing surface or solution are still needed.

**Figure 1:**
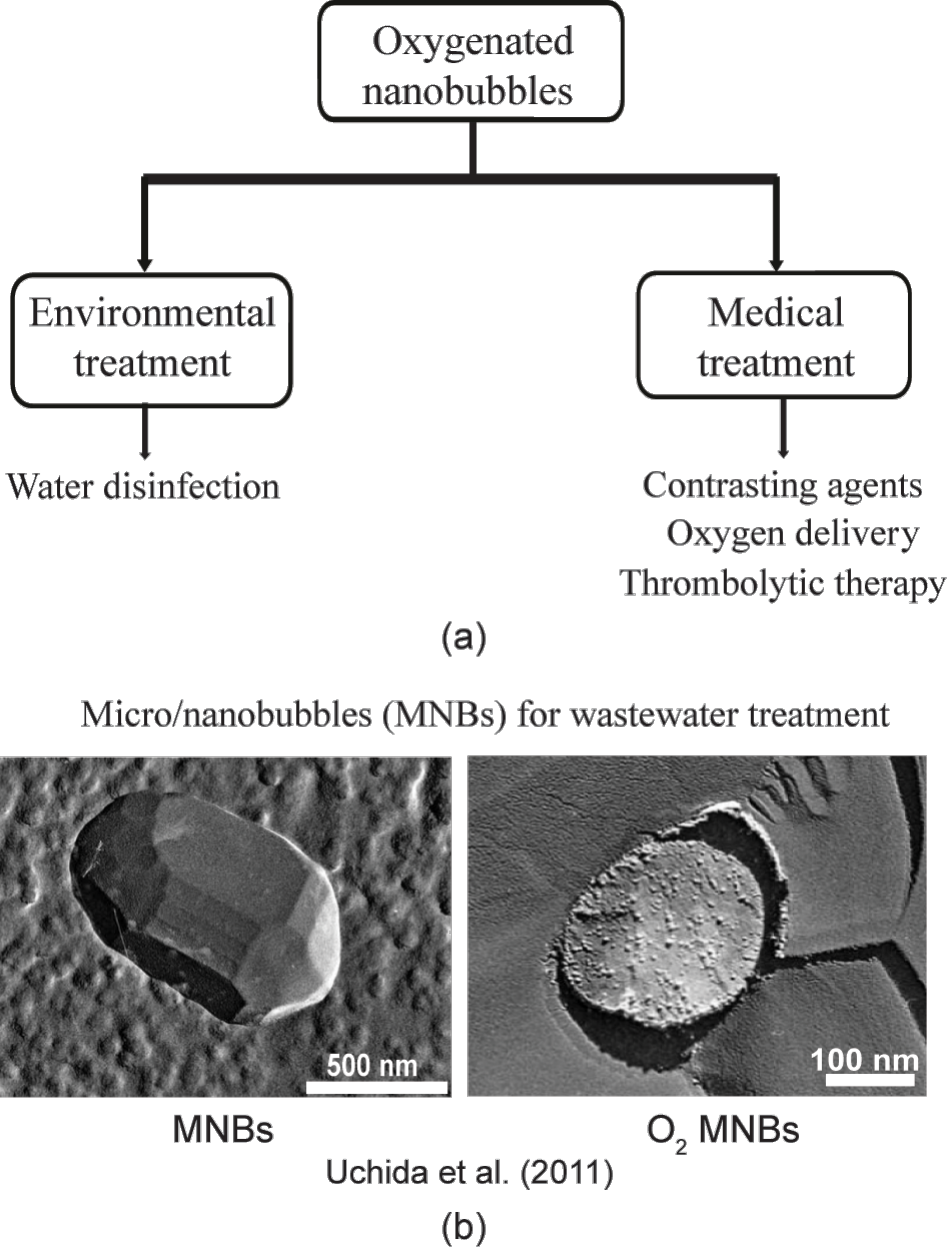
(a) Flow chart showing some applications of oxygenated nanobubbles. Reproduced with permission from [55]. Copyright (2013) Elsevier. (b) An exemplary application of nanobubbles containing oxygen in wastewater treatment, reproduced with permission from [51], Copyright (2011) Uchida et al. under a Creative Commons Attribution License.

Nanobubbles, which are bubbles with dimensions of 5–100 nm in height and 50–800 nm in diameter at the interface of solid and liquid, were found on some hydrophobic and superhydrophobic surfaces [14,16,32–38]. The nanobubbles change the interface of solid and liquid and therefore are believed to affect the drag of liquid flow. Due to the high Laplace pressure of the nanobubbles, the bubbles should theoretically disappear after several milliseconds [39]. However, it is found that nanobubbles can be stable for hours [40] or days [41]. The nanobubbles gained much attention due to the abnormal stability and many potential applications [42–51]. Figure 1a shows some applications of oxygenated nanobubbles, and Figure 1b shows an example, the transmission electron microscopy (TEM) images of oxygenated micro/nanobubbles which are applied for wastewater treatment. Furthermore, nanobubbles at the interface of solid and liquid are believed to be the source of the boundary slip [10,52–54]. On the other hand, some researchers show that the boundary slip is a result of the interaction of solid and liquid molecules [12,15], which means that the existence of nanobubbles on the interface is not a necessary condition for boundary slip. The study of nanobubbles and boundary slip with an applied voltage can be an important evidence for the discussion of the relationship between nanobubbles and boundary slip.

**Figure 2:**
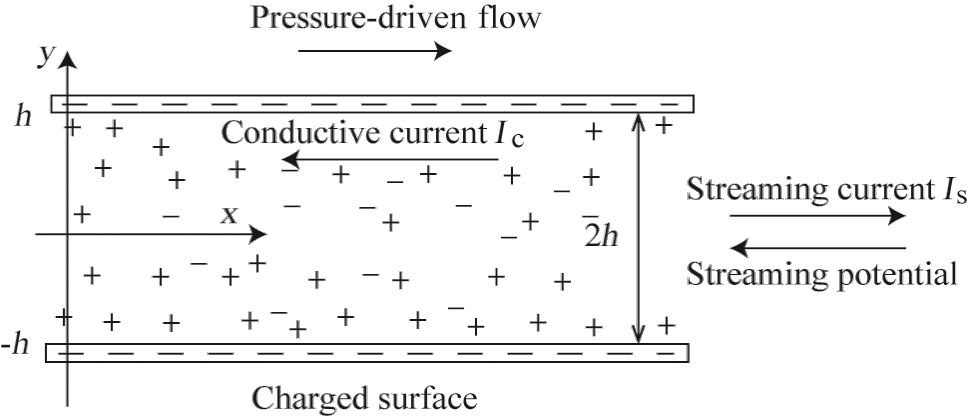
Schematic of the model of a channel formed by two infinitely parallel surfaces separated with a distance of 2*h*.

When a liquid contacts a solid surface, the solid surface will be charged as a result of the adsorption of ions or of protonation/deprotonation. The charged solid surface will lead to a layer in which ions are strongly attracted to the charged surface and do not move because of electrostatic interactions. Furthermore, there is another layer close to the surface called diffuse layer which is composed of ions that are attracted to the surface charge by Coulomb force. This structure is called electrical double layer (EDL). Because of the EDL a streaming potential [56–57] and a streaming current will be generated during when a pressure-driven liquid flow is passing by the solid–liquid interface (Figure 2). The induced streaming potential will apply an electrical force on the liquid in the opposite direction of the flow resulting in a decrease of the flow velocity. The electrical force, which is related to the streaming potential and electrical conductivity of the flow, can be considered a drag force [58–62]. It is also believed that the surface charge density has a relationship with nanobubbles and boundary slip [59,63].

Generally speaking, with an applied voltage, the surface properties and the interaction of the surface and liquid will be affected. In particular, the wettability, surface charge, nanobubbles and boundary slip are correlated with each other and applied voltage. As a result, the drag of liquid flow in micro scale can be affected by applied voltage. The study of surface wettability, surface charge, nanobubbles and boundary slip with applied voltage may provide a method to reduce the drag of liquid flow in micro/nano scale.

In this review, recent studies on the effect of an applied voltage on surface wetting, nanobubbles at the interface, and boundary slip are summarized. Firstly, the influence of an applied voltage on the contact angle (CA) and contact angle hysteresis (CAH) on a polystyrene (PS) surface is discussed, followed by the study of the nanobubbles on a PS surface with applied voltage. The influence of applied voltage on the surface charge density and boundary slip on an octadecyltrichlorosilane (OTS) surface is discussed as well. The relationship between nanobubbles at the interface and boundary slip length is discussed based on the changes of nanobubbles and slip length with applied voltage.

This review is divided into four sections. In section 1, the influence of an applied voltage on the CA and CAH of deionized (DI) water and saline on PS surface will be discussed. In section 2, the experimental results of nanobubbles studies with applied voltage in different solutions will be introduced in detail. The mechanism of the influence of applied voltage on nanobubbles will be discussed based on the study of the surface charge density with applied voltage. In section 3, the influence of the surface charge density on the liquid flow will be discussed. The experimental results of boundary slip studies with applied voltage will be reported. Based on these experimental results the relationship between nanobubbles and boundary slip will be discussed as well. Finally in the last section, a summary and an outlook are provided.

## Review

### 1 Contact angle and contact angle hysteresis with applied voltage

When a voltage is applied to a droplet deposited on a solid surface, the surface tension between solid and liquid changes, which leads to a change of the wettability of the surface, the so called electrowetting [10–11,64–65]. The surface tension between solid and liquid decreases with increasing applied voltage, leading to a decrease of the CA. The change of the CA with the applied voltage *V* can be expressed by the Young–Lippmann equation [64,66] as:

[1]
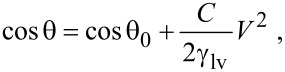


where θ_0_ is the original CA in the absence of an voltage, *C* is the capacitance of the dielectric layer, and γ_lv_ is the surface tension between liquid and vapor.

In 1875, Lippmann found the electrowetting phenomenon and presented the Young–Lippmann equation, which is acknowledged as the basic of the electrowetting theory. In 1993, Berge added a dielectric layer between the liquid and solid [67] and greatly reduced the electrolysis that hindered the development of the applications of electrowetting. The electrowetting on dielectrics (EWOD) opened a new age for the study of electrowetting, the EWOD has been studied theoretically and experimentally [10,65]. Applications based on EWOD have been presented [68–74].

**Figure 3:**
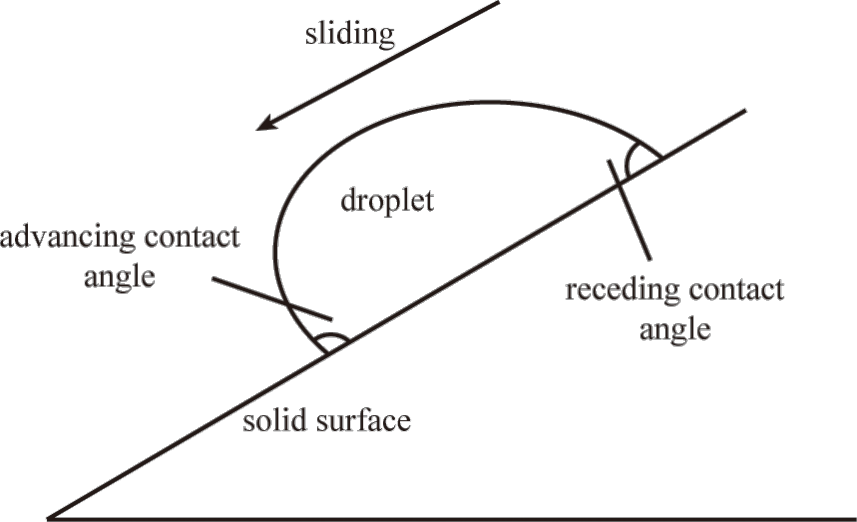
Schematic showing advancing and receding contact angle. Contact angle hysteresis is equal to the difference of advancing and receding contact angle.

Besides CA, CAH is another important parameter to describe the wettability of the surface. CAH is the difference between advancing CA and receding CA, as shown in Figure 3. When the droplet slides on a solid surface, the CAH of the droplet will affect the drag. Generally speaking, it is easier to move the droplet with smaller CAH. Li and Mugele found the CAH of saline on Teflon (AF1600) could be decreased by applying an alternating current (AC) voltage [75]. Bhushan and Pan found the CAH of DI water on a PS surface could be decreased by applying an AC voltage [76].

The study of the effect of applied voltage on CA and CAH on PS surface is the basis of this review. An effective method of applying a voltage to the system is introduced. DI water and saline were used. To explain the trend of the experimental data with DC voltage, a model has been presented.

#### 1.1 Experimental setup

PS pellets with a molecular weight of 35,000 (Sigma-Aldrich) were dissolved in toluene (Mallinckrodt Chemical) in a sonication bath for one hour to obtain the PS solution with a concentration of 1% (w/w). The PS surface was prepared as coating on a p-type boron doped silicon wafer (1–20 Ω·cm, Silicon Quest International) substrate, which was coated with a 300 nm thick silicon oxide layer. Before coating the PS film on the substrate, the wafers was cleaned in a sonication bath of acetone first, and then cleaned in a sonication base of isopropyl alcohol. The PS film was then obtained by spin coating with the prepared PS solution at a speed of 2000 rpm. After that, the PS coated wafer was placed in an oven at 53 °C for 4 h to remove the remaining solvents. The thickness of the PS coating measured by an ellipsometer (model L116C, Gaertner Scientific Corporation) was found to be 66 nm. A DI water droplet was deposited on the spin coated PS surface and the CA of the DI water was measured to be about 94° by using an automated goniometer (Model 290, Rame-Hart Instrument Corporation). The prepared PS surfaces are thus considered to be hydrophobic. To apply the voltage to the system, the sample was glued to a metal sample plate with conductive silver paint, in this case, the silicon substrate and the sample metal plate were electrically connected and the voltage could be applied. The metal sample plate was used as an electrode. A stainless steel wire was used as another electrode by inserting it into the droplet.

**Figure 4:**
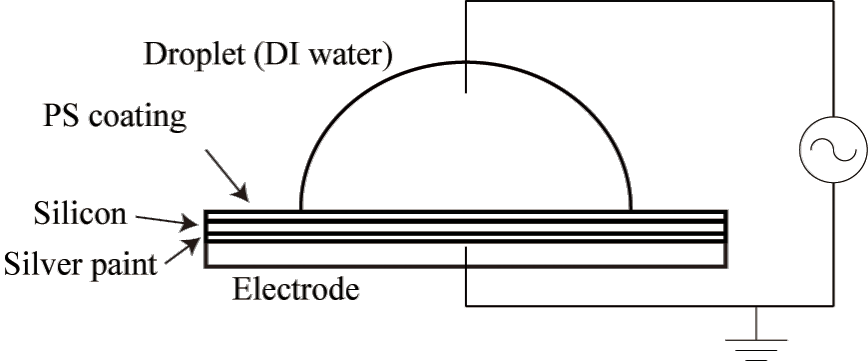
Schematic of the experimental setup used for the measurement of the CA as a function of applied voltage. Reprinted with permission from [76]. Copyright (2011) American Chemical Society.

The experimental setup is shown in Figure 4. The applied voltage was generated by a power amplifier (model 50, Ling-Altec Electronics Inc.) which amplifies an electrical signal generated by a function generator (33120A, HP). Both DC voltage and AC voltage were applied. The AC voltage was in square wave, and the frequency was varied from 0.5 to 10^4^ Hz. A high-speed camera (FS100, Canon) with 1152 × 864 pixels was used to observe the shape of the droplet, and the image was recorded. For each picture, the CA was measured five times by using Scion Image on a PC. To measure the CAH of the droplet, the droplet volume was inflated by a syringe. Before the contact line moves, the maximum value of the CA was recorded as the advancing CA. Then the droplet was sucked in by the syringe. Before the contact line moves, the minimum value of the CA was recorded as the receding CA. The CAH was calculated as well. The whole experimental process was limited to 10 min in order to minimize the effect of evaporation.

In the experimental setup, there are several layers such as silver paint, silicon, PS coating and DI water. The interface of the layers will have a tendency to be electrostatically charged. When the applied voltage has an opposite polarity, the charging is additive. As a result, there will be a discharge current [77] which may lead to a damage of the brittle PS coating. To avoid the damage of the sample, the applied AC voltage was biased with a minimum value of 0 V.

#### 1.2 Model for the DC electrowetting

As mentioned earlier, when a voltage *V* is applied between the substrate and the droplet, the decreased CA of the droplet can be expressed by the Young–Lippmann equation (Equation 1). The dielectric layer in this work has two layers, one is the 300 nm thick silicon oxide layer, and the other is the PS film. Then the equivalent capacitance of the system can be expressed as [76]:

[2]
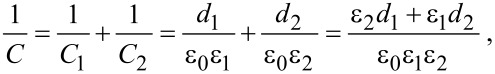


where *C*_1_, *C*_2_ are the capacitances of silicon oxide layer and PS film, ε_1_, ε_2_ are the relative dielectric constants of the silicon oxide and PS, *d*_1_, *d*_2_ are the thickness of silicon oxide layer and PS film, respectively, and ε_0_ is the dielectric constant of the vacuum. Combining Equation 1 and Equation 2, one gets

[3]
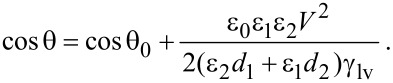


Here we define an electrowetting coefficient *k* for this work,

[4]
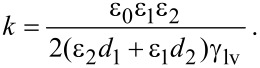


Then the equation can be expressed as,

[5]
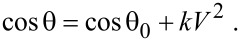


The electrowetting coefficient *k* describes the sensitivity of the system to the applied voltage, and a larger value of *k* means that the CA will change more with same applied voltage. In this work, the interface of silicon and PS film will be charged due to the different work function. The charge layer may reduce the effective voltage. As a result, the experimental value of the electrowetting coefficient *k* will be smaller than the theoretical calculated value based on the known value of ε_0_, ε_1_, ε_2_, *d*_1_, *d*_2_ and γ_lv_ [76].

#### 1.3 Effects of applied voltage on the CA and CAH of DI water droplet on PS surface

DC and AC voltages, respectively, were applied to the system. With DC voltage, the CA and CAH were measured and recorded as functions of the changing voltage. In the AC case, the CA were measured and recorded as functions of different frequencies and peak-to-peak voltages. When the frequency of the applied AC voltage is lower than 10 Hz, the oscillation of the droplet was visible, then the maximum and minimum of the CA were recorded to describe the oscillation. Due to the influence of the oscillation at frequencies lower than 10 Hz, the CAH with applied AC voltage was measured at frequencies higher than 10 Hz.

**Figure 5:**
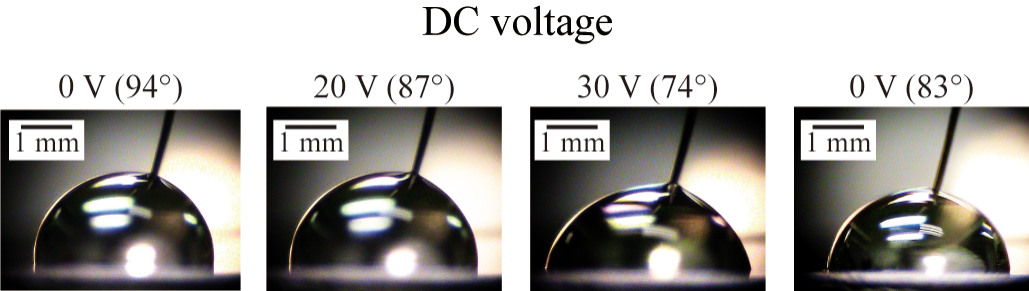
DC voltage dependence of the shape and CA of the droplet. The voltage was varied from 0 to 30 V, and then back to 0 V (the right image). Reprinted with permission from [76]. Copyright (2011) American Chemical Society.

**Figure 6:**
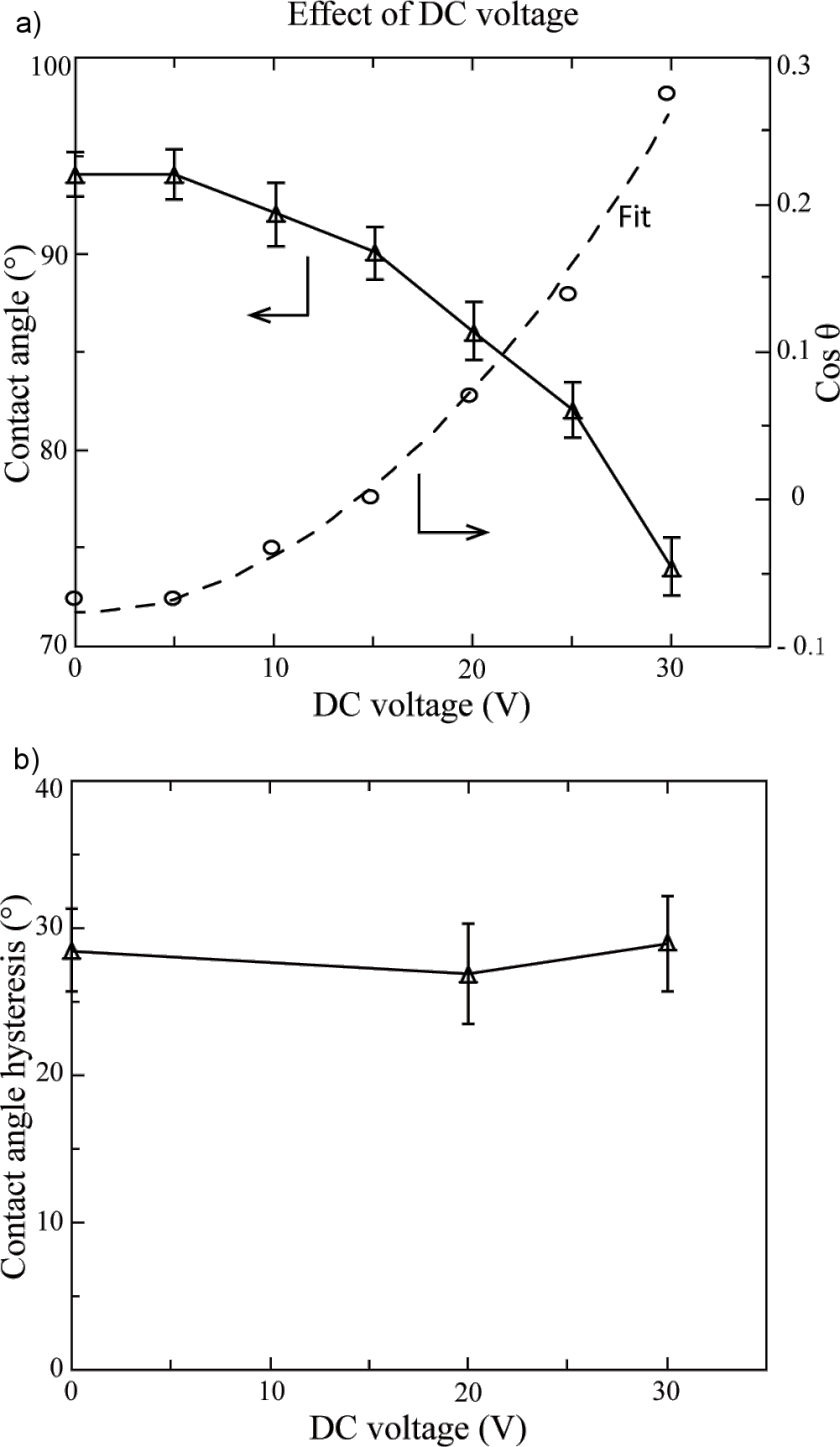
(a) The CA of the droplet and its cosine value as functions of applied DC voltage. Equation 5 was used to fit the cosine value of the CA. (b) The CAH of the droplet as a function of applied DC voltage. The error bars represent one standard deviation ±σ. Reprinted with permission from [76]. Copyright (2011) American Chemical Society.

**1.3.1 DC voltage effects on the CA and CAH on PS surface:** The images of the droplet of DI water on PS surface with different DC voltages are shown in Figure 5. The voltage was increased from 0 to 30 V and then was removed. The CA was measured for each voltage and it decreased from 94° at 0 V to 74° at 30 V. When the applied voltage was removed, the CA increased to 83°, instead of the original CA of 94°. The reason of this observation is believed that after the voltage decreased to 0 V, there was a residual electric field because of the capacitive system. Figure 6 shows the CA as a function of the applied DC voltage. The cosine values of the CA with each voltage are also shown in Figure 6. The data was fitted by Equation 5 to obtain the electrowetting coefficient *k*, which is 3.26 × 10^−4^ V^−2^, the *R*^2^ value was 0.99. The calculated value of *k* was also obtained by using Equation 4 as 6.49 × 10^−4^ V^−2^ for comparison. The calculated value of *k* is larger than the experimental fitted value of *k*, which is in agreement with the previous discussion.

**Figure 7:**
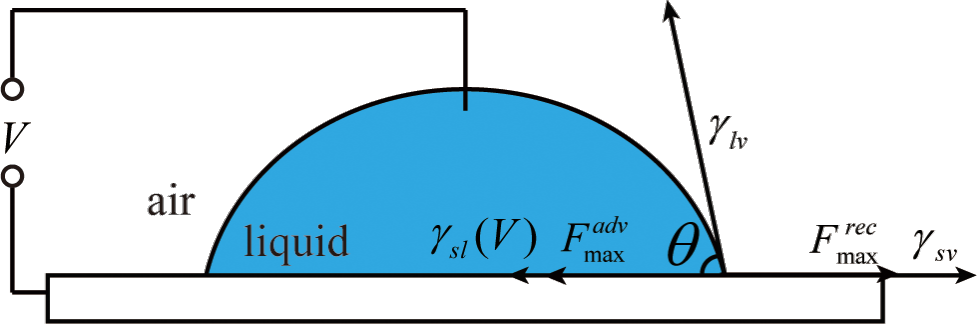
Schematic of the balance of forces at the contact line between surface and water with applied voltage *V*. γ_sl_(*V*) is the voltage dependent surface tension between solid and liquid, γ_sv_ and γ_lv_ are the solid–vapor and liquid–vapor interfacial tension, respectively.

The CAH with different applied voltages are shown in Figure 6. The change of CAH with applied voltage is not obvious. An analysis of the balance of horizontal forces at the unit length of the contact line is carried out to understand this observation. The pinning force on the contact line is assumed to have a same value at any position but opposite directions for advancing and receding. As shown in Figure 7 the balance of horizontal forces at the unit length of the contact line can be expressed by the following:

[6]
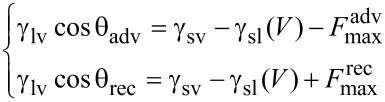


where θ_adv_ and θ_rec_ are the advancing and receding CA, 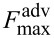 and 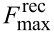
are the pining force for advancing and receding, respectively.

When a voltage *V* is applied, γ_sl_(0) will decrease to γ_sl_(*V*). Assuming that γ_lv_, γ_sv_, and the pinning forces *F*_max_ remain constant with the applied voltage, then the change of the advancing and receding CA can be expressed as

[7]
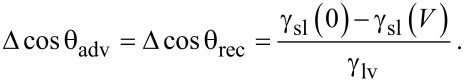


That means with applied voltage, both cos θ_adv_ and cos θ_rec_ decrease to the same extent. In this experiment, with a same change in the cosine value, the change of CA will be also similar. As a result, the change of CAH is unobvious [76].

**Figure 8:**
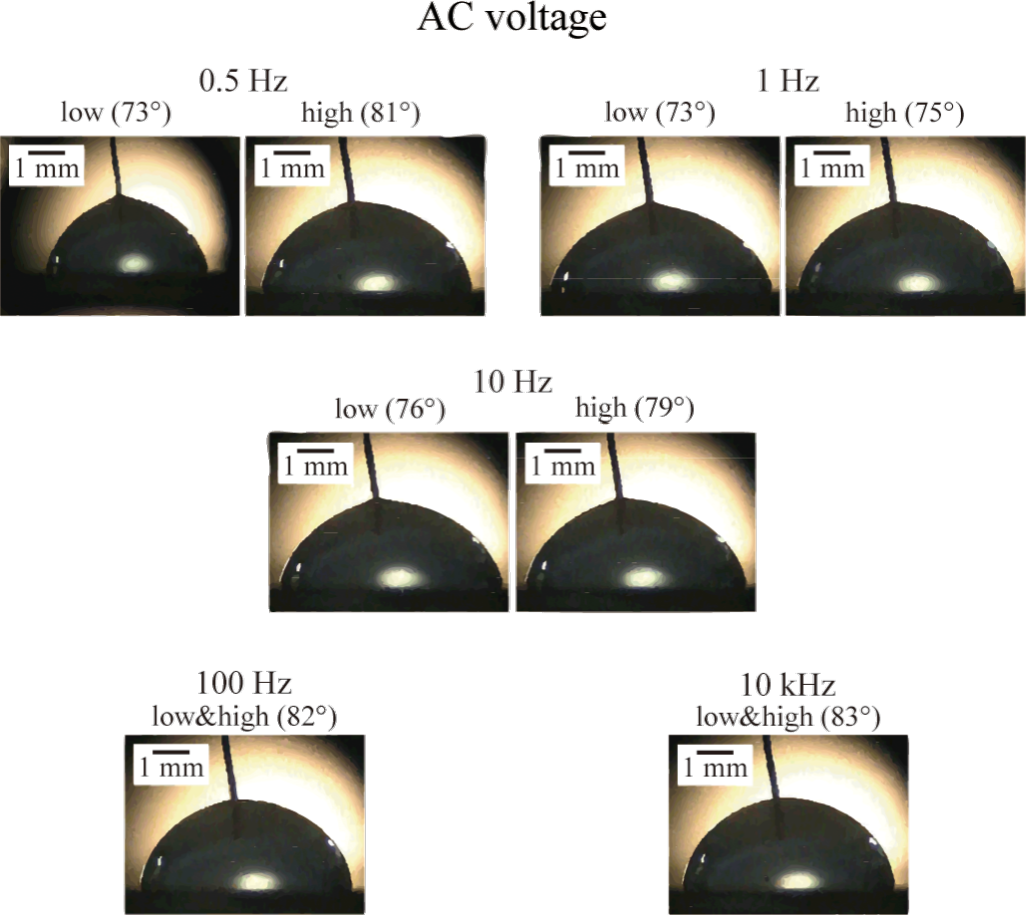
Frequency dependence of the shape and CA of the droplet. The peak-to-peak value of the AC voltage is 30 V, and the minimum value of the AC voltage is 0 V. At lower frequency (lower than 10 Hz), the droplet is oscillating, two different contact angles for one frequency were recorded; one is the droplet with maximum CA labeled high and the other is the droplet with minimum CA labeled low. Reprinted with permission from [76]. Copyright (2011) American Chemical Society.

**Figure 9:**
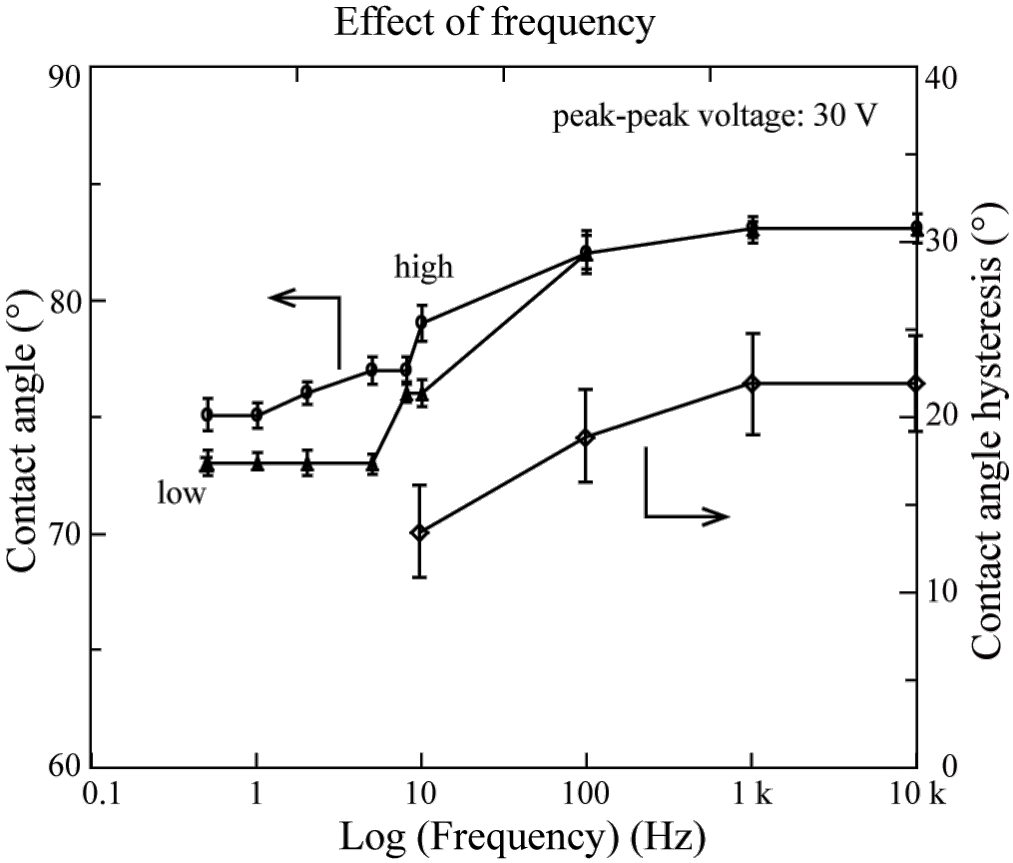
The CA and CAHs of the droplet as function of the frequency of an AC voltage with a peak-to-peak value of 30 V. The frequency is in log scale. The “high” and “low” plots mean the maximum and minimum value of the CA, respectively. Reprinted with permission from [76]. Copyright (2011) American Chemical Society.

**1.3.2 AC voltage effects on the CA and CAH on a PS surface:** When an AC voltage was applied, the droplet was oscillating. At low frequency (<10 Hz), the oscillation of the droplet was recordable. Two images for each frequency of the applied AC voltage are used to describe the oscillation: one is the droplet with the maximum CA, the other is the droplet with the minimum CA. Figure 8 shows the images of the droplet with different frequencies of the applied AC voltage with a peak-to-peak voltage value of 30 V. The maximum CA of the droplets are labeled high, and the minimum CA of the droplets are labeled low. Figure 9 shows the measured CA as a function of the frequency of the applied AC voltage. Both the high CA and the low CA remain constant, followed by a steady increase above a frequency of about 1 Hz [76].

The CAH as a function of the frequency is also shown in Figure 9. It is obvious that the CAH with AC voltage is smaller than the CAH with DC voltage and it increases with an increasing frequency, comparable to the static CA. The balance of horizontal forces at the unit length of the contact line can be used to explain this phenomenon: The applied AC voltage is switching between 0 and 30 V, so the surface tension between solid and liquid is also switching between the maximum value and minimum value based on Equation 6. The advancing CA should be obtained when the surface tension between solid and liquid reaches the maximum value while the receding CA should be obtained when the surface tension between solid and liquid reaches the minimum value. The advancing CA will have a similar trend with the AC voltage as the static CA, which means the advancing CA will increase with the increase of frequency. The receding CA, however, should be always obtained when the voltage is equal to 0 V. As a result, the receding CA will remain constant with an AC voltage. With a smaller advancing CA and the same receding CA, the CAH with applied AC voltage should increase with increasing frequency and should be smaller than the initial value which is in agreement with the experimental results.

#### 1.4 Summary

In this section, the influence of an applied voltage on the CA and CAH of DI water on a PS surface is studied experimentally. With an increasing applied DC voltage, the CA of the droplet on PS surface decreased. By removing the applied voltage, the CA did not increase back to the initial value. To explain the experimental result, a theoretical model was developed and discussed. When an AC voltage was applied, with lower frequency, the oscillation of the droplet was recorded with maximum and minimum value of CA. The maximum and minimum value of CAs increased or remained constant at low frequency, then increased with the increasing frequency of applied voltage. The measured CAH with AC voltage was smaller than with DC voltage and increased as static CA with the increasing frequency of applied AC voltage.

The electrowetting on the PS surface is the subject of the following studies. The surface tension changes with an applied voltage at the macro scale. A simple assumption is that the changed surface tension will affect the size and distribution of nanobubbles, and then affect the drag of fluid flow. The study of the CAH with applied AC voltage provides a method to decrease the drag of moving droplet.

### 2 Nanobubbles at the interface of solid and liquid with applied voltage

Nanobubbles at the interface of solid and liquid are of interest due to their wide applications. As an example, nanobubbles can be used for imaging and drug monitoring [50] and for drug delivery [44]. Nanobubbles containing oxygen, in particular, have possible applications in medical and environmental treatment [47,49]. Nanobubbles are also believed to affect the drag of fluid flow [10] and have a relationship with boundary slip [53,78].

**Figure 10:**
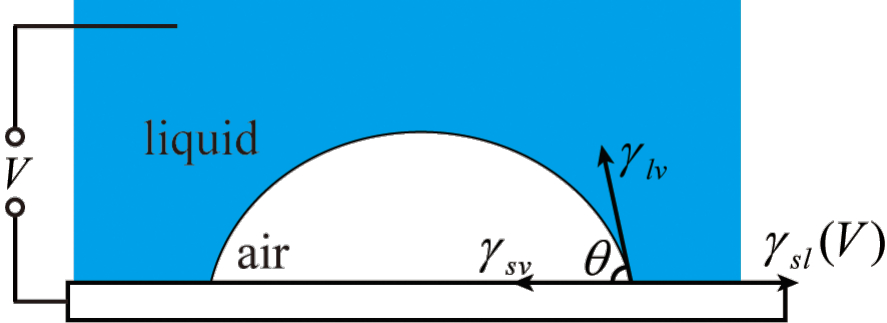
Schematic of the balance of forces of a nanobubble at the contact line between surface and water with applied voltage *V*. γ_sl_(*V*) is the voltage dependent surface tension between solid and liquid, γ_sv_ and γ_lv_ are the solid–vapor and liquid–vapor interfacial tension.

The study of nanobubbles with applied voltage can provide a method to control the size and distribution of nanobubbles and also can help to understand the properties of nanobubbles. As mentioned in the last section, the surface tension changed with applied voltage. The balance of horizontal forces at the unit length of the contact line of nanobubbles is shown in Figure 10. With applied voltage, the surface tension between solid and liquid will be smaller, which should lead to a decrease of the covered area of the nanobubbles and an increase of the contact angle, θ. However, nanobubbles are believed to be negatively charged due to the pressure of hydroxy ions in aqueous solutions [63]. The electric field provided by the applied voltage will affect the charged nanobubbles. Thus, the effect of the applied voltage on nanobubbles at the interface of solid and liquid should be studied experimentally.

Currently, most of the nanobubble researches have focused on DI water or saline solutions. Few reports have focused on fluids containing oxygen. Considering the potentially wide applications of nanobubbles in oxygenated fluid, two types of oxygenated solutions were used in the study.

#### 2.1 Experimental setup

**Figure 11:**
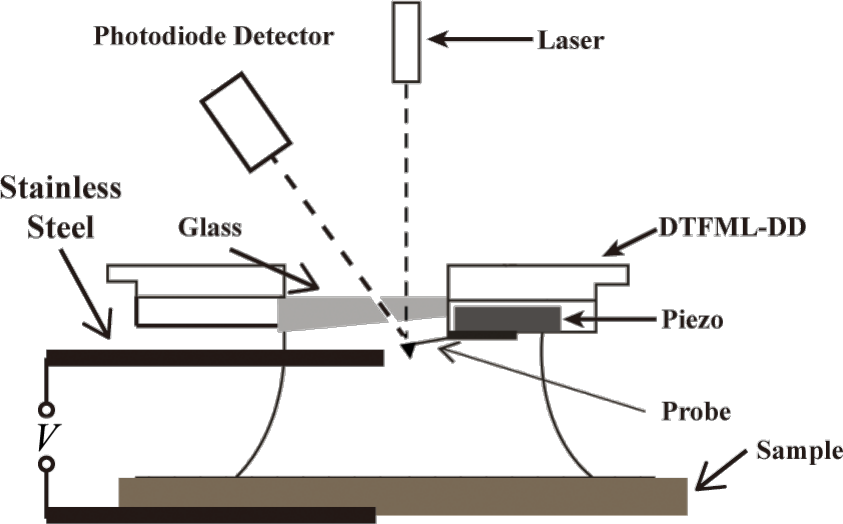
Schematic of the experimental setup used for imaging nanobubbles with applied voltage.

The sample was prepared as described in the last section. The nanobubbles were imaged and recorded with a Dimension 3000 atomic force microscope (AFM) (Bruker Instruments, Santa Barbara, CA) in tapping mode. In order to image in liquid, the AFM cantilever was held by a polychlorotrifluoroethylene (PCTFE) fluid cell cantilever holder (DTFML-DD), which has a piezo to drive the cantilever in tapping mode when working in fluids. A silicon nitride cantilever PNPTR (Nanoworld, Neuchâtel) was used for tapping mode. The tip radius was smaller than 15 nm, the stiffness was 0.32 N·m^−1^, and the cantilever is Cr/Au backside coated. The tip was driven at a frequency close to the resonance frequency in liquid. Images were taken with 512 scan lines at a scan rate of 2 μm/s. To minimize the force on the nanobubbles, the setpoint was set to 95% of the free amplitude. All data presented were obtained under ambient conditions (temperature of 22 ± 1 °C, humidity of 45–55% RH). The voltage was applied as shown in Figure 11. For each applied voltage, at least three images at different areas were recorded to confirm the scanning itself did not result in the change of the nanobubbles.

Besides DI water, three kinds of saline were used in study. First one is normal saline solution (sodium chloride (NaCl) 8000 mg/L, potassium chloride (KCl) 200 mg/L, sodium phosphate dibasic (Na_2_HPO_4_·7H_2_O)_2_, 160 mg/L, potassium phosphate monobasic (KH_2_PO_4_) 100 mg/L) (Invitrogen, Grand Island, NY). Another two oxygenated saline solutions (Revalesio Corporation, Tacoma, WA) were PNS60 and RNS60. PNS60, which is referred to as saline 1, in this paper contained 0.9% saline and excess oxygen. Electrokinetically altered oxygenated saline RNS60, which is referred to as saline 2 in this paper, contained 0.9% saline and 50–60 ppm oxygen without active pharmaceutical ingredients [79].

To understand the influence of the applied voltage on the nanobubbles, a pretreated PS surface was used to study the relationship between the nanobubbles and the surface charge density. The PS surface was treated by an antistatic gun first. Negative ions were deposited on the surface. To study the change of the surface charge density before and after treatment, the electrostatic force was measured by AFM [80]. A colloidal probe with a sphere glued on the top of normal probe was used. The diameter of the sphere was measured as 134 μm. When the sphere was driven towards the surface in solution at a low velocity, such as 0.22 μm/s, the hydrodynamic force is small and can be ignored and the measured force can be considered as electrostatic force.

#### 2.2 Images of nanobubbles in various solutions with applied voltage

Experimental results revealing the morphology, size and density of the nanobubbles at the interface of PS surfaces in various solutions using AFM will be discussed. The influence of an applied voltage on the property of nanobubbles in various solutions will be described. An explanation will be given.

**Figure 12:**
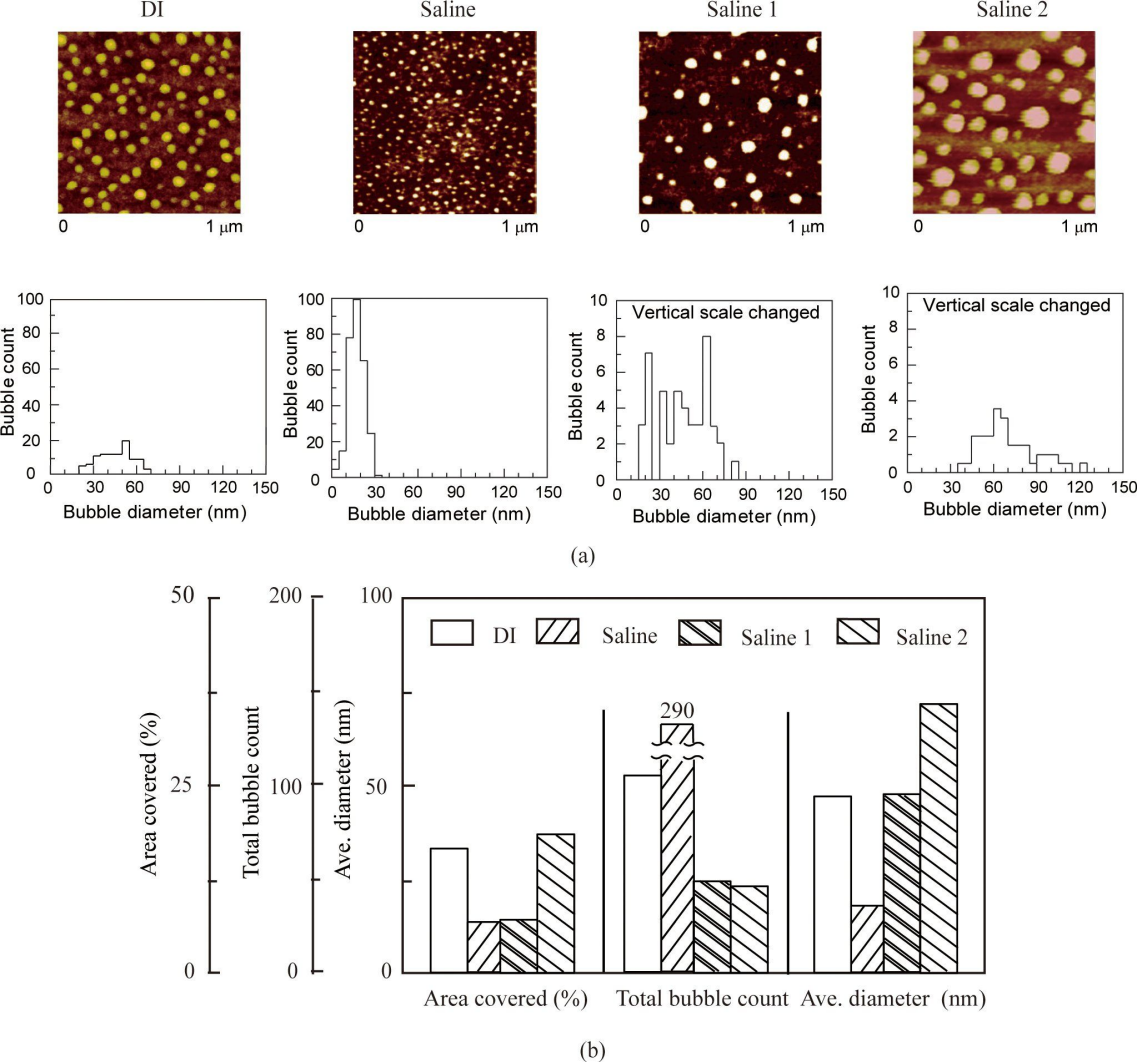
Nanobubbles in DI water, saline, saline 1 and saline 2 on PS surface imaged by using AFM. (a) Height images and corresponding histograms show the nanobubbles produced in oxygenated and electrokinetically altered fluids (saline 1 and saline 2) to be bigger compared with DI water and saline. (b) Summary of the area covered, total count and average diameter of the nanobubbles data shown in (a). Saline 1 and saline 2 shows larger and fewer nanobubbles on the PS film compared with DI and saline. Reproduced with permission from [55]. Copyright (2013) Elsevier.

**2.2.1. Morphology of nanobubbles in various solutions:** The AFM topographic (height) images and corresponding histograms of nanobubbles in DI water, saline, saline 1 and saline 2 are shown in Figure 12a.

It is shown that saline 1 and saline 2 solutions produced larger but fewer spherical features as compared to DI water and saline. The images were analyzed with the commercial SPIP software. Figure 12b shows the geometrical analysis of the nanobubbles in DI water and three kinds of salines. For the nanobubbles in DI water, saline, saline 1 and saline 2, the percentage of the nanobubbles covered area is approximately 18%, 8%, 9%, and 20%; the average diameter of the nanobubbles was 45 nm, 17 nm, 47 nm and 71 nm; the total count of nanobubbles in the range of 1 μm × 1 μm was 105, 290, 46 and 41, respectively. The percentage of the nanobubbles covered area in saline 2 is the highest while the total count of the nanobubbles in saline is the highest. The average diameter of nanobubble in saline 1 and saline 2 were found to be larger.

It has been reported that nanobubble formation on hydrophobic surfaces is dependent on the gas saturation of the liquid [81–82]. Van Limbeek and Seddon reported the dramatic effect of the gas type on the nanobubble size [83]. The increase of oxygen content can be the reason for the increase in the average diameter of nanobubbles in saline 2 over saline 1 over saline.

**Figure 13:**
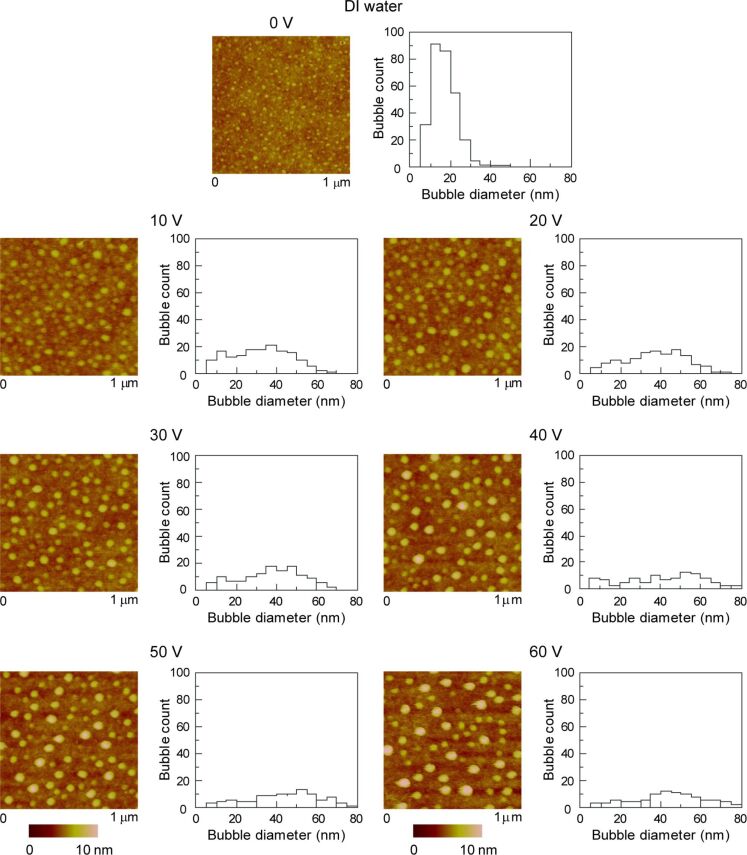
Height images and corresponding histograms of the size distribution of nanobubbles on PS surface with applied voltage in DI water. Reproduced with permission from [55]. Copyright (2013) Elsevier.

**Figure 14:**
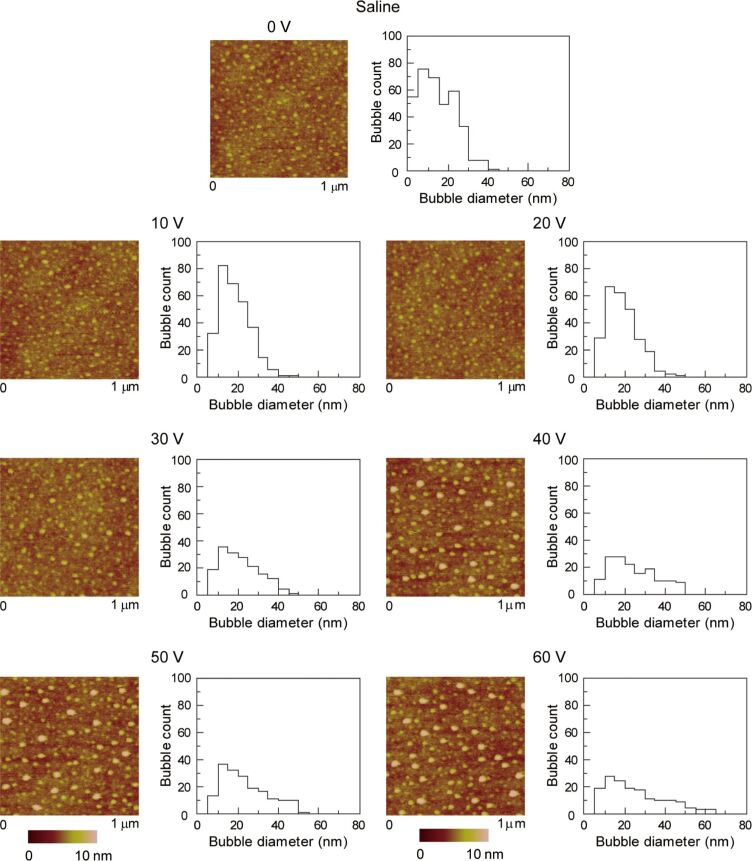
Height images and corresponding histograms of the size distribution of nanobubbles on PS surface with applied voltage in saline. Reproduced with permission from [55]. Copyright (2013) Elsevier.

**Figure 15:**
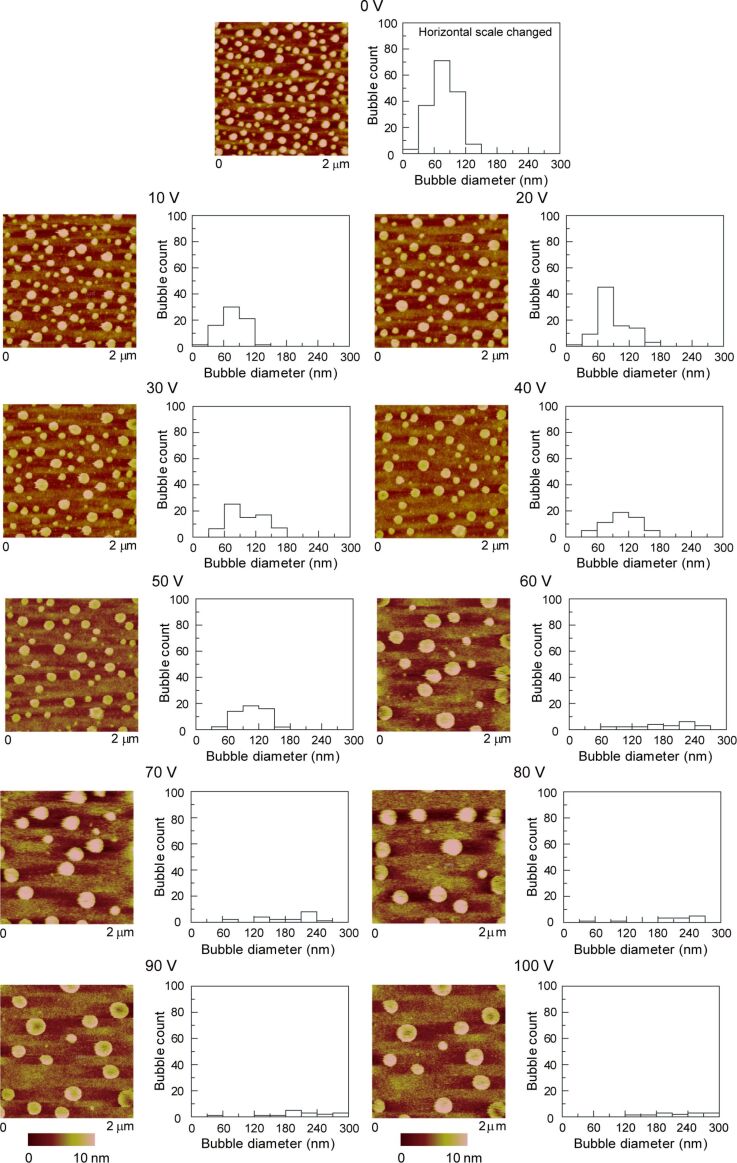
Height images and corresponding histograms of the size distribution of nanobubbles on PS surface with applied voltage in saline 2. Note that scan size is 2 μm × 2 μm to capture large bubbles as compared to scan size 1 μm × 1 μm in Figure 13 and Figure 14. Reproduced with permission from [55]. Copyright (2013) Elsevier.

**Figure 16:**
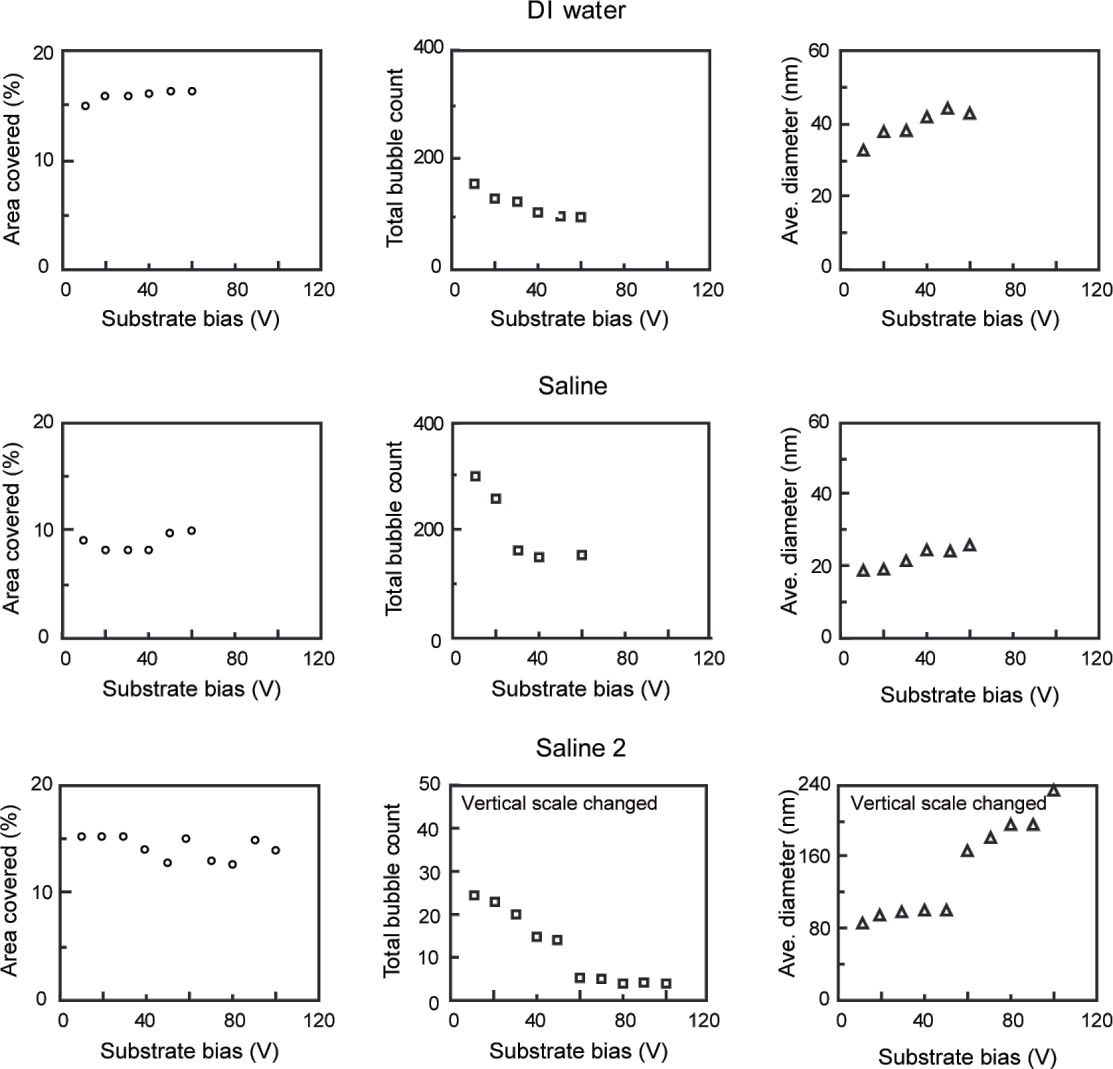
Geometrical distribution of nanobubbles as a function of applied voltage on PS surface in DI water (first row), saline solution (second row) shown in Figure 13 and Figure 14, respectively, and in saline 2 (third row) shown in Figure 15. Reproduced with permission from [55]. Copyright (2013) Elsevier.

**2.2.2. Morphology of nanobubbles with applied voltage:** DI water, saline and saline 2 were used to study the effect of applied voltage, salt concentration and gas saturation on nanobubbles. Figure 13, Figure 14 and Figure 15 show the height images of nanobubbles on a PS surface with applied voltage in DI water, saline and saline 2, respectively. In DI water and saline, the voltage range was 0–60 V, at voltages larger than 60 V, damage of the surface occurred. In saline 2, the voltage range was 0–100 V. Histogram analyses of the images are also shown in Figure 13, Figure 14 and Figure 15. It is obvious that, in every kind of solution, with an increasing positive voltage applied to the substrate, the total count of nanobubbles decreased and the average diameter of nanobubbles increased. The quantitative estimate of the area covered with nanobubbles, the total count of nanobubbles, and the average diameter of the nanobubbles with applied voltage in DI water, saline and saline 2 are shown in the first, second and third row of Figure 16, respectively. Due to the different image scale for saline 2, which is 2 μm ×2 μm in Figure 15, compared with the image scales for DI water and saline, which are 1 μm ×1 μm in Figure 13 and Figure 14, the data of total count of nanobubbles in saline 2 was firstly obtained from Figure 13c and then divided by four in order to compare with the data in DI water and saline.

The area covered by nanobubbles increases in DI water and saline while there is no obvious change in saline 2 with increasing positive voltage to the substrate. The total count decreases from about 300 to 92 in DI water while the decrease in saline was from about 300 to 150 and in saline 2 it was from about 25 to 3. The diameter increases from 18 nm to 44 nm in DI water while the increase in saline was from around 18 nm to 25 nm and in saline 2 it was from around 84 nm to 227 nm. In DI water and saline 2, there is an about 2.5 times increase in average diameter of nanobubbles while an 1.4 times increase in average diameter of nanobubbles in saline.

With applied voltage, the nanobubbles extended instead of shrink which shows an opposite trend compared to electrowetting at the macro scale. A possible explanation for this effect could be the following. An electric field is generated through the applied voltage. The negatively charged nanobubbles were pulled by the electric field towards the surface, leading to an increase in the diameter. At the meantime, the aggregation of some of the extending nanobubbles led to a further increase of the average diameter. This aggregation is less likely in saline, in which the nanobubbles are smaller than in DI water and saline 2, resulting in a smaller increase in average diameter of the nanobubbles, which is in agreement with the experimental result. The aggregation may lead to the discontinuous increase of the average diameter of nanobubbles in saline 2, which has a small total count of nanobubbles at a voltage of 60 V (Figure 15) [55].

**Figure 17:**
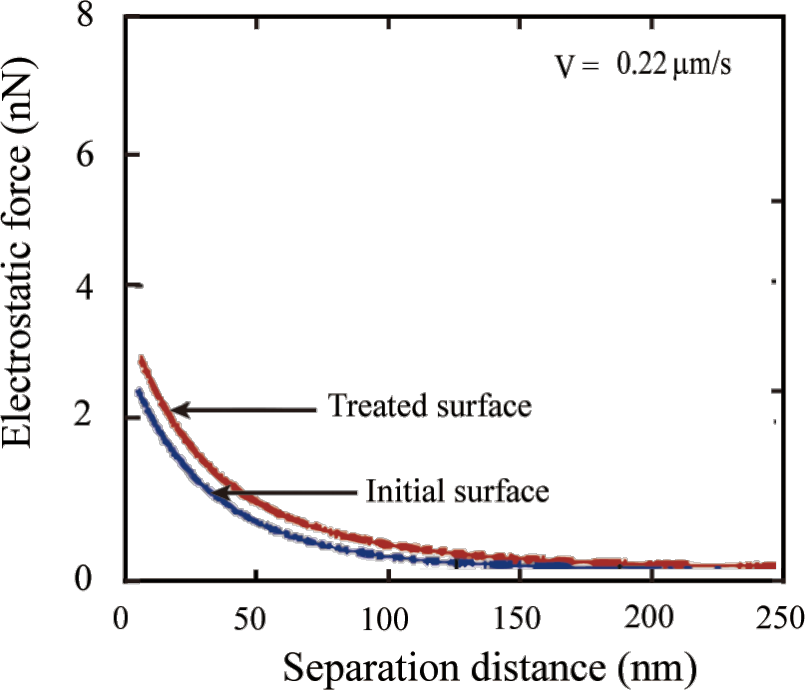
Electrostatic forces on initial PS surface and pretreated PS surface in DI water.

The experiment on the pretreated PS coated surface may support this explanation. Figure 17 shows the measured electrostatic force on the glass colloidal probe before and after treatment. It is obvious that after treatment the electrostatic force increased, which means the surface charge density on the PS coated surface increased.

When a glass surfaces is in contact with water, it will acquire a charge, which can be described by [84]: SiOH = SiO^−^ + OH^+^. As a result, the glass surface of the colloidal probe is negatively charged in water. Since the repulsive electrostatic force was measured between the colloidal probe and the PS surface in water, the PS surface can also be believed to be negatively charged.

**Figure 18:**
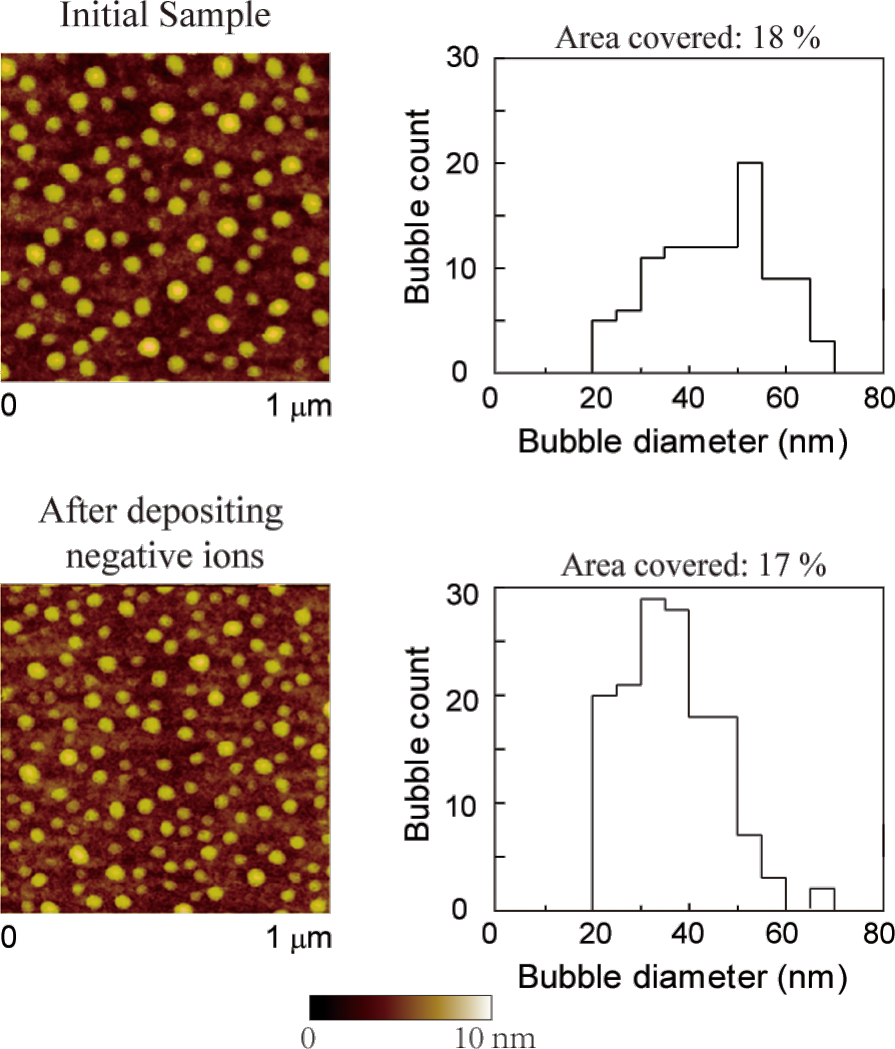
Height images and corresponding histograms of the size distribution of nanobubbles on the initial PS surface and the pretreated PS surface in DI water. Reproduced with permission from [55]. Copyright (2013) Elsevier.

The image of nanobubbles on initial PS coated surface and treated PS coated surface are shown in Figure 18, the histogram analyses of the images are shown as well. It is obvious that on the treated PS coated surface, the total count of nanobubbles increases while the average diameter of nanobubbles decreases. The coverage of nanobubbles on the treated PS coated surface is smaller than the coverage of nanobubbles on the normal PS coated surface. Since oxygen is poorly soluble in water, when the DI water is deposited on the treated surface, the water molecules will catch the electrons on oxygen ions, and the oxygen will be releases into the air. The coverage of the nanobubbles did not increase on the treated surface, which can be an evidence that the nanobubbles on the treated surface did not contain the released oxygen.

Both the nanobubbles and the PS surface are negatively charged. Increasing the surface charge density may lead to an increase of the electrostatic repulsion force between the nanobubbles and surface, which might result in a decrease of the nanobubble diameter and a decrease of the probability of the aggregation of nanobubbles, which will finally lead to the decrease of the average diameter and the increase of the total count of nanobubbles. This experiment result may support the explanation for the change of nanobubbles with applied voltage.

#### 2.3 Summary

The formation of nanobubbles on the PS surface was observed in different solutions with an AFM. Nanobubbles were imaged in DI water, saline, saline 1, and saline 2. The diameter, total count and covered area of the nanobubbles on the PS surface were a function of the salt and gas concentration. As shown in the AFM images, larger and fewer nanobubbles were obtained in saline 1 and saline 2, which contained oxygen, compared with that in DI water and saline.

Applying a voltage to the PS surface led to an increase of the average diameter of nanobubbles and a decrease of the total count of the nanobubbles in DI water, saline and saline 2. The effects of the applied voltage on the nanobubbles are more pronounced in DI water and saline 2 which have larger bubbles. It can be an explanation that the charged nanobubbles were pulled by the electric filed provided by the applied voltage. By applying negative ions on the surface, in DI water, the surface charge density increased. In the meantime, the average diameter of the nanobubbles decreased and total number of nanobubbles increased. The electrostatic repulsion force between the nanobubbles and the surface may be enhanced by depositing electrons on the surface, as a result, the average diameter of nanobubbles may decrease, which may support the explanation.

### 3 Boundary slip and surface charge with applied voltage

As stated in the introduction, boundary slip condition and surface charge density can affect the drag and be affected by the applied voltage. In this section, the boundary slip on a hydrophobic surface will be measured with applied voltage. The surface charge density will be studied by measuring the electrostatic force on a colloidal probe. The influence of EDL on the drag of liquid flow will be introduced. Unlike the experiment of wettability and nanobubbles, the boundary slip and surface charge were studied on an OTS surface, which has a higher contact angle (106°) and a larger boundary slip. On the PS surface, which has a CA of 94°, the slip length is too small to be measured by the experimental setup.

#### 3.1 Experimental setup

To prepare the OTS surface, a silicon wafer (Silicon Quest International) with 300 nm thick thermally grown silicon oxide coating was used as the substrate. First, the wafers were immersed in piranha solution, which is a 3:1 mixture of sulfuric acid and 30% hydrogen peroxide, for 30 min. Second, the wafers were rinsed with water and ethanol for several times and dried by compressed air. Then the wafers were immersed into a 1% (v/v) OTS (SIO6640.1, Gelest) solution in anhydrous toluene for 24 h. Finally, to remove the unabsorbed molecules from the surface, the wafers were copious rinsed with toluene and dried by compressed air. The CA of DI water on the OTS surface was measured as 106 ± 2° by automated goniometer.

**Figure 19:**
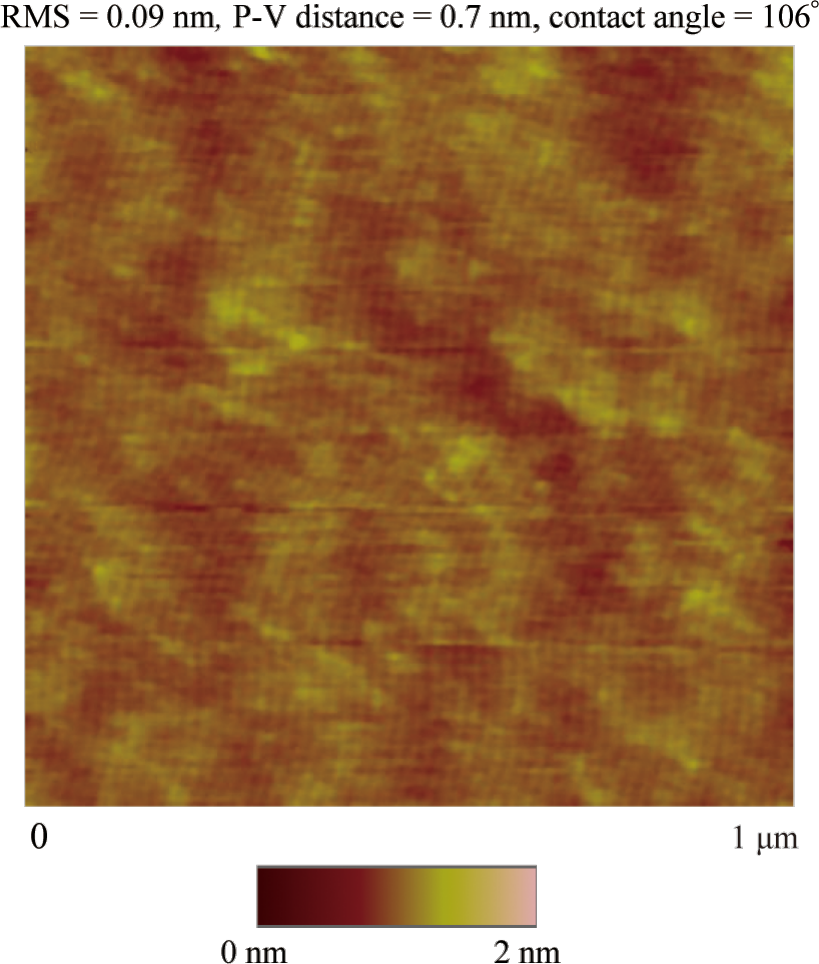
AFM Images, surface roughness and contact angle data of OTS surface in air. Reproduced with permission from [94]. Copyright (2013) Elsevier.

The process for applying a voltage to the system was the same as described in section 3. The surface was imaged in air with an AFM first. Figure 19 shows an image of the OTS surface in air obtained by AFM. The RMS value was 0.09 nm and the P–V distance was 0.7 nm. The slip length were measured by an AFM in contact mode [85] with a colloidal probe. The diameter of the borosilicate sphere (GL018B/45-33, MO-Sci Corporation) which was glued on the top of a rectangular cantilever (ORC8, Bruker) is 59.5 μm.

**Figure 20:**
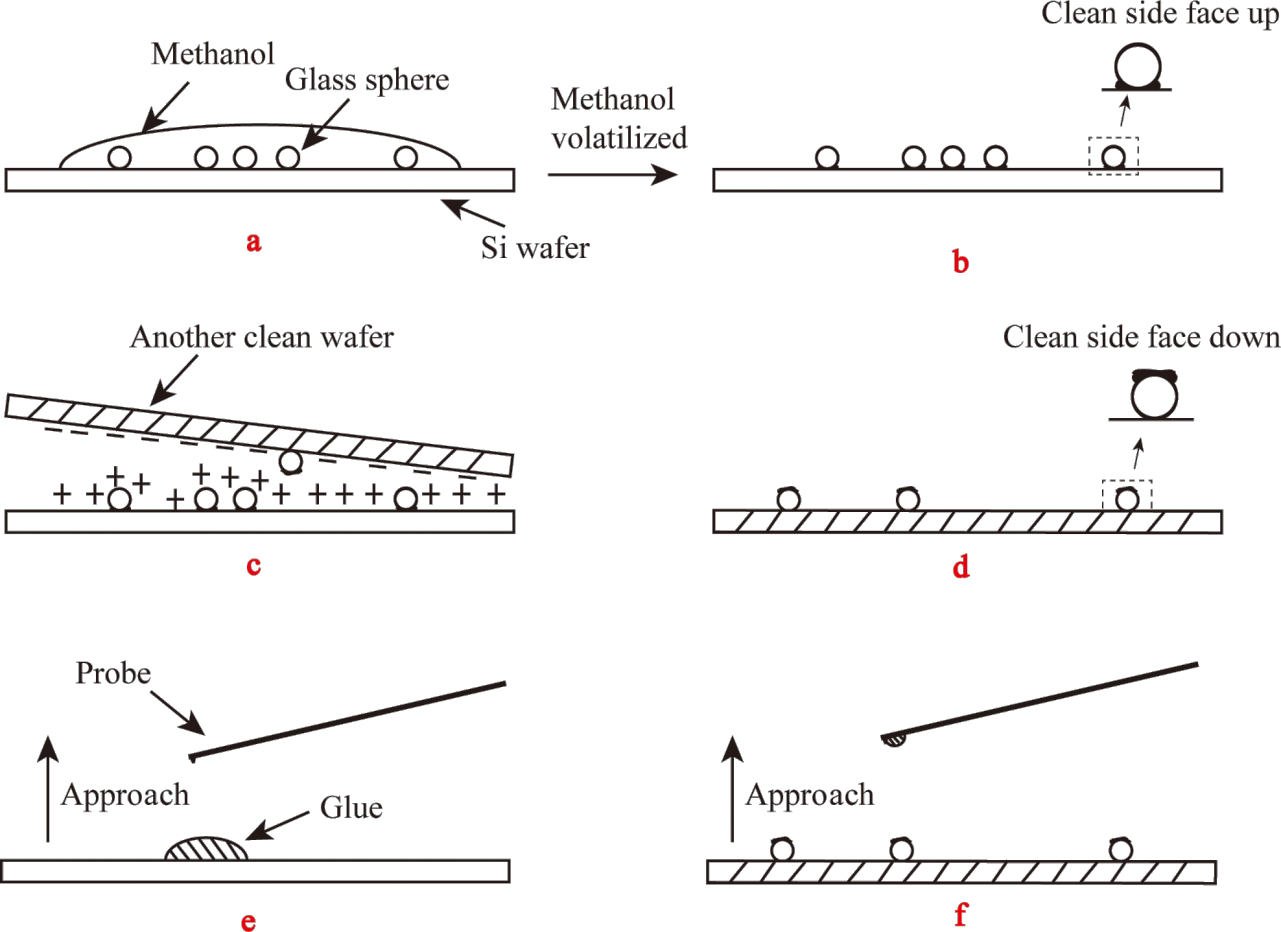
Process of making clean colloidal probe (described in the text).

The process for making the colloidal probe was performed with an optical microscope (Optiphot-2, Nikon). The spheres were firstly cleaned in a sonication bath of acetone. Then they were cleaned in a sonication bath of methanol for several times and the cleaned spheres were kept in a bottle of methanol solution. The methanol solution was stirred and then several milliliters of the solution was dropped on a piece of cleaned wafer as shown in Figure 20a. After evaporation of the methanol, the clean part of the spheres on the wafer faced up (b). The spheres on the wafer were positively charged by an antistatic gun (ZEROSTAT 3), another clean silicon wafer was negatively charged (c). The negatively charged wafer was driven to approach the spheres, and some of the spheres were attached to the charged wafer by electrostatic force with the dirty part facing up (d). A small amount of glue was deposited on a glass slide. The cantilever slowly approached the glue until the glue is transferred to the end of the cantilever (e). Then the cantilever slowly approached on of the sphere prepared in step d until the sphere was attached (f). This process of making colloidal probe can make sure the clean part of the sphere is facing down when used by AFM.

To measure the slip length, the sample was driven by an AFM approaching to the probe at a fixed velocity in liquid and the force on the probe was recorded as a function of separation distance. The slip length can be obtained by analyzing the recorded force. The slip length on a clean flat hydrophilic surface, such as silicon surface, was always firstly measured as a reference.

DI water and saline solution were used in this experiment. The saline solution was described in the experimental part of section 3. On a flat hydrophilic surface, such as silicon surface, the deflection of the colloidal probe, *Def*, in the experiment can be given as [86]:

[8]
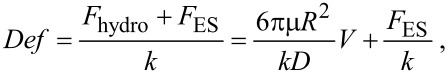


where *F*_hydro_ is the hydrodynamic force, *F*_ES_ is the electrostatic force, μ is the dynamic viscosity of the liquid, *V* and *R* are the velocity and radius of the sphere, respectively, *D* is the separation distance, and *k* is the stiffness of the cantilever.

The deflection of the cantilever can be obtained by the AFM. For slip length measurement, the driving velocity was 77 μm/s. Then the measured force should include both of hydrodynamic force and electrostatic force. The electrostatic force is independent of the velocity, which means that with a decreasing driving velocity, the electrostatic force remain constant while the hydrodynamic force decreases. If the velocity is small enough, such as 0.22 μm/s, the hydrodynamic force will be smaller than 0.1 nN and can be ignored compared with the electrostatic force. As a result, the measured force at low velocity can be considered as electrostatic force only. The hydrodynamic force was obtained by subtracting the obtained electrostatic force from the high-velocity data. Measurements were always made with both high driving velocity (77 μm/s) and low driving velocity (0.22 μm/s) on the same sample.

To obtain the hydrodynamic force and electrostatic force, the value of *k* is also needed. By fitting the cantilever deflection data as a function of the separation distance on a hydrophilic silicon surface using Equation 8, the value of *k*/(6·π·μ·*R*^2^) was obtained as 16.2 nm·s. With the measured value of *R* = 29.75 μm and the known value of μ = 9.8 × 10^−4^ kg/m·s for water at room temperature [87], the stiffness *k* can be calculated as 0.267 N/m.

For flat surfaces, which can be assumed to have boundary slip, the ratio of velocity for approaching over the hydrodynamic force on the sphere can be expressed, when D >> b, as [26]:

[9]
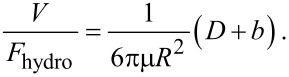


The position at which the linear extrapolation of the curve *V/F*_hydro_ (D >> b) intercepts the separation distance axis gives the slip length *b*.

#### 3.2 The influence of surface charge density on the boundary slip and drag of liquid flow

It is believed that the boundary slip can be explained by the forces between the molecules of solid and liquid. The charge existing on the surface provides another interaction between molecules of solid and liquid beside the Lennard-Jones potential, so the boundary slip will be affected by the surface charge. Barrat and Bocquet give a Green–Kubo expression for the interfacial friction coefficient [13]:

[10]
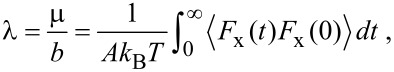


where μ is the viscosity of the liquid, *A* = *L*_x_*L*_y_ is the area of the solid surface under consideration and *F*_x_ is the Ox component of the instantaneous force exerted by the surface on the liquid at equilibrium. The total force can be separated as: *F*_x_ = *F*_LJ_ + *F*_ES_. It can be infer that the boundary slip will be reduced with the existing of surface charge. Joly et al. analyzed the relationship between the boundary slip and surface charge density [15], the slip length with a surface charge density of Σ is:

[11]
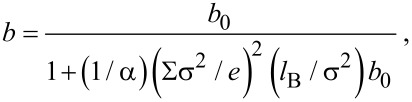


where *b*_0_ is the slip length without considering surface charge, *l*_B_ is the Bjerrum length, σ is solid molecular diameter, α ≈ 1 is a numerical factor, *e* = 1.6 × 10^−19^ C. Note here that there is an error made by Joly et al. [15], they missed a factor of 1/σ at the second item in the denominator. It has been corrected in Equation 11, which shows that the slip length will decrease when the surface charge density increases.

Besides slip length, the surface charge density is also believed to affect the velocity of the liquid flow by producing a streaming potential and then an electrical force on the pressure-driven flow. To investigate the effect of EDL on the flow with boundary slip condition, a model of one-dimensional channel with two parallel surfaces is developed (Figure 2). Pressure-driven flow is considered. To simplify the analysis, the slip length is considered as a fixed value.

When the surface is charged, the net charge in the fluid should be equal to the surface charge on the solid surface, the relationship between surface charge density Σ and the net charge density of liquid ρ_e_ can be written as:

[12]
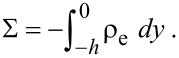


Then the potential ψ can be expressed by the Poisson equation [88]:

[13]
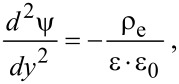


where ε is the relative dielectric constant of the liquid, ε_0_ is the permittivity of vacuum. The boundary conditions of Poisson equation are

[14]
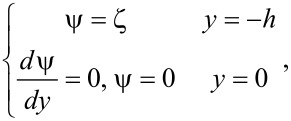


where ξ is the surface potential at the boundary, called zeta potential. The distance between two surfaces is assumed to be large enough that the EDL will not overlap with one another. Since the surface charge density is difficult to measure, the zeta potential is always used to describe the amount of surface charge. If the surface charge density is assumed to depend on the properties of the fluid and surface, rather than the channel size and the flow direction, the relationship between Σ and ξ can be expressed as [88–89]:

[15]
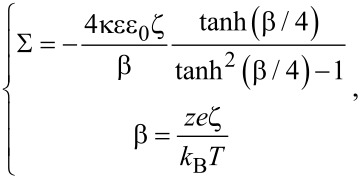


where κ is the Debye length, *k*_B_ is the Boltzmann constant, *T* is the absolute temperature, *z* is the chemical valence of ions, *e* is the elementary charge. The net charge density ρ_e_ can be expressed by using a Boltzmann distribution as [90]:

[16]
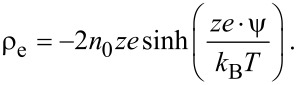


For a pressure-driven flow, when considering the electrical force exerted on the flow by the EDL, the flow can be described by a modified Navier–Stokes equation as [91]:

[17]
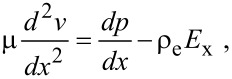


where μ is the dynamic viscosity of the fluid, *v* is the velocity of the fluid, *dp*/*dx* is the pressure gradient used to drive the flow, *E*_x_ is the streaming electrical field produced by the EDL. The boundary conditions of velocity for the Navier–Stokes equation are

[18]
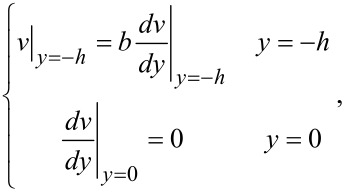


where *b* is the slip length which is assumed to be a constant.

In a steady state, the net electrical current should be zero, so the relationship between streaming current, *I*_S_, and conduction current, *I*_C_, is [91]:

[19]
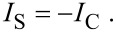


The streaming current *I*_S_ can be expressed as:

[20]
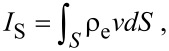


where *S* is the cross-sectional area of the channel and the conduction current *I*_C_ can be expressed as:

[21]
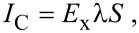


where λ is the electrical conductivity of the fluid. The electrical field can be given as

[22]
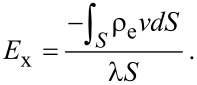


Then the modified Navier–Stokes equation can be rewritten as

[23]
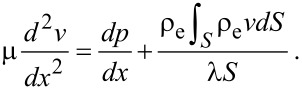


Combining Equation 14, Equation 16 and Equation 23, we can get:

[24]
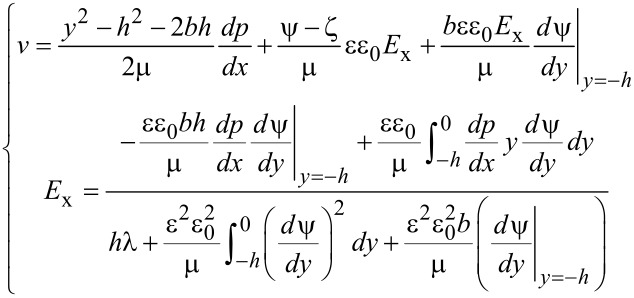


and

[25]
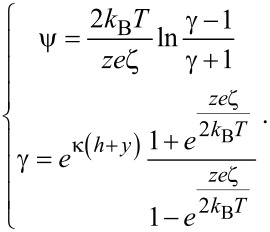


Next we define the volumetric flow rate as

[26]
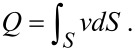


**Figure 21:**
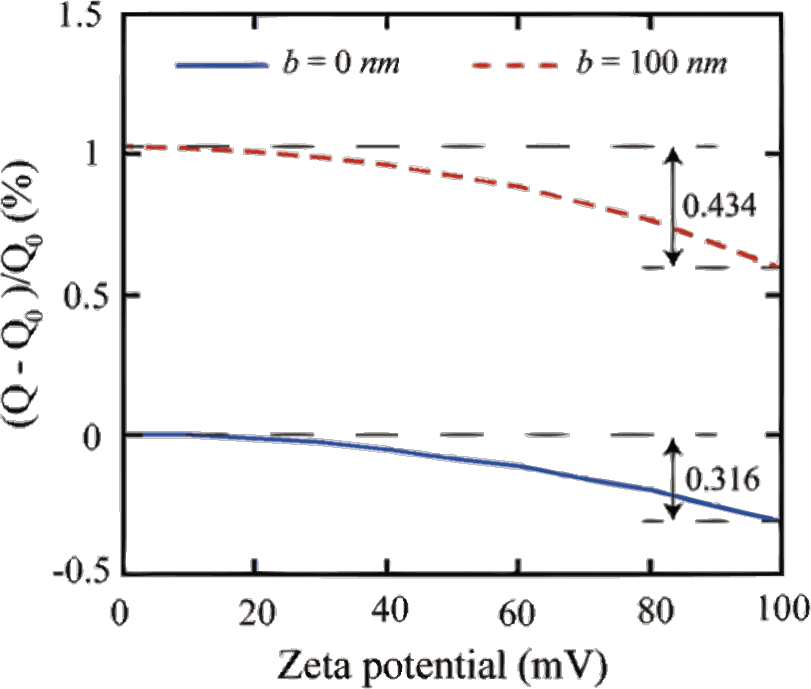
The effect of EDL on the volumetric flow rate. *Q*_0_ is the flow rate without considering the slippage and EDL, and *Q* is the flow rate considering EDL and for the solid line *b* = 0 nm; for the dashed line *b* = 100 nm. The zeta potential is varied from 0 mV to 100 mV as an increasing surface charge, 0 mV is equal to not considering EDL.

In this study, DI water, which can be considered as a 1:1 symmetric electrolyte solution, is used. We assumed *n*_0_* =* 6.02 × 10^19^/m^3^, which corresponds to an electrolyte with a concentration of 1 × 10^−7^ mol/L. For a temperature of *T* = 293 K, we get μ *=* 1.002 × 10^−3^ Pa·s, ε *=* 80.20, ε_0_ = 8.854 × 10^−12^ F/m [87] and λ = 4.19 × 10^−6^ S/m [92]. For the purpose of analysis, we assumed *dp/dx* = −1 × 10^6^ N/m^3^ and *b* = 100 nm. With Equation 24 to Equation 26, the relationship between the volumetric flow rate with and without consideration of EDL is shown in Figure 21. The results show that with considering EDL, the volumetric flow rate decreases with increasing zeta potential. When there is a slip, the effect of EDL on the drag is even larger.

When a voltage is applied to the system, the surface charge density will be changed. With a changed surface charge density, the velocity of the liquid flow will be changed by the streaming potential and changed boundary condition.

#### 3.3 Boundary slip and surface charge density with applied voltage

**Figure 22:**
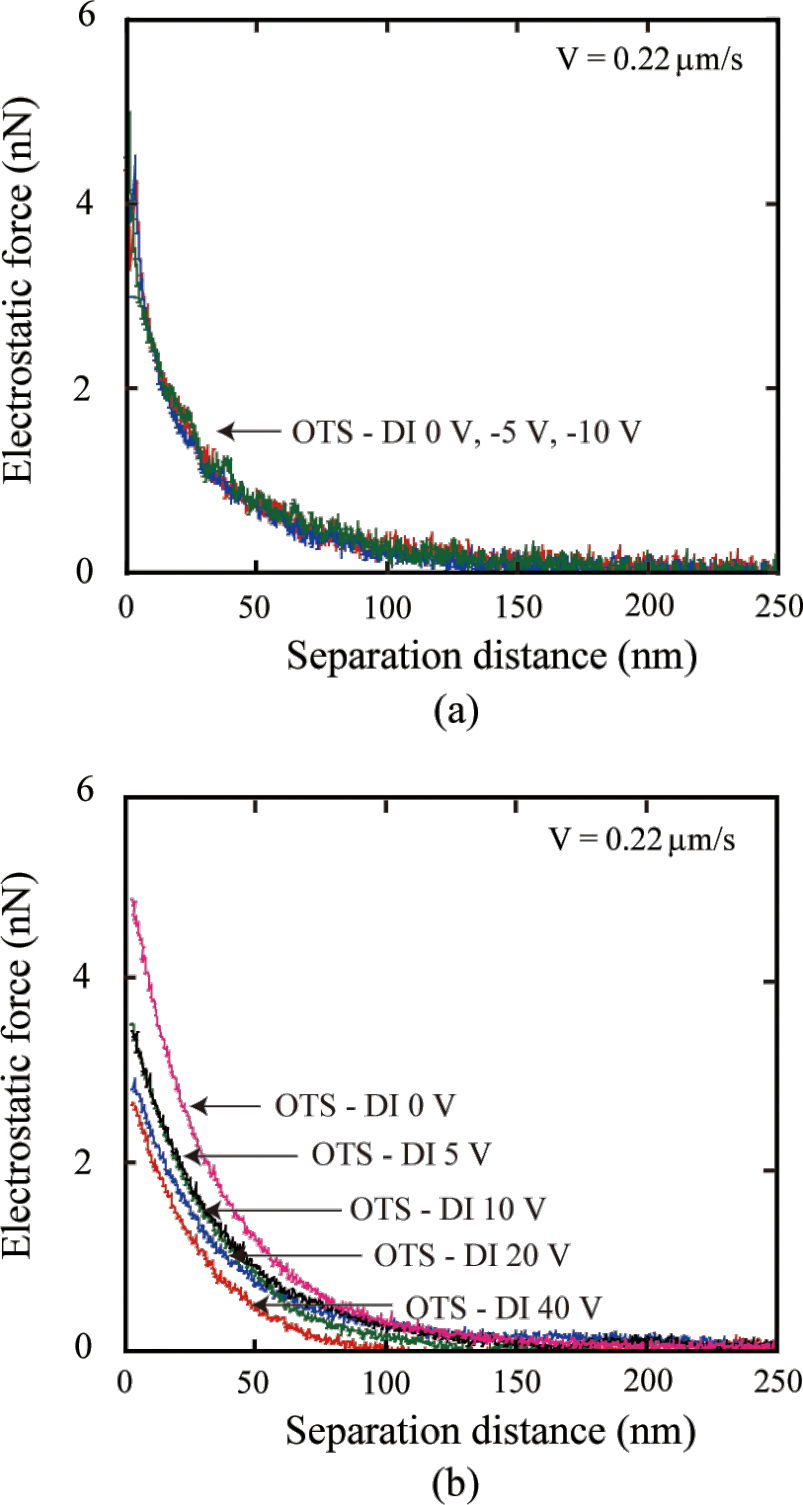
(a) Electrostatic force in DI water with different negative voltages, −5 V and −10 V, applied to the substrate. (The sample was destroyed at higher voltages). (b) Electrostatic force in DI water with different positive voltages, 0–40 V, applied to the substrate. (The sample was destroyed at higher voltages). Reproduced with permission from [94]. Copyright (2013) Elsevier.

**3.3.1 DI water:** The change of surface charge density with applied voltage is analyzed by measuring the electrostatic force on the probe with applied voltage. The measured electrostatic force in DI water as a function of separation distance with negative and positive applied voltages to the substrate are shown in Figure 22. With an increasing positive voltage applied to the substrate, the electrostatic force decreased while it remained constant with an increasing negative applied voltage to the substrate. Based on the experimental setup, a lower electrostatic force on the probe shows a lower surface charge density on the OTS surface. Thus, applying positive voltage to the substrate can reduce the surface charge density on OTS surface in DI water.

**Figure 23:**
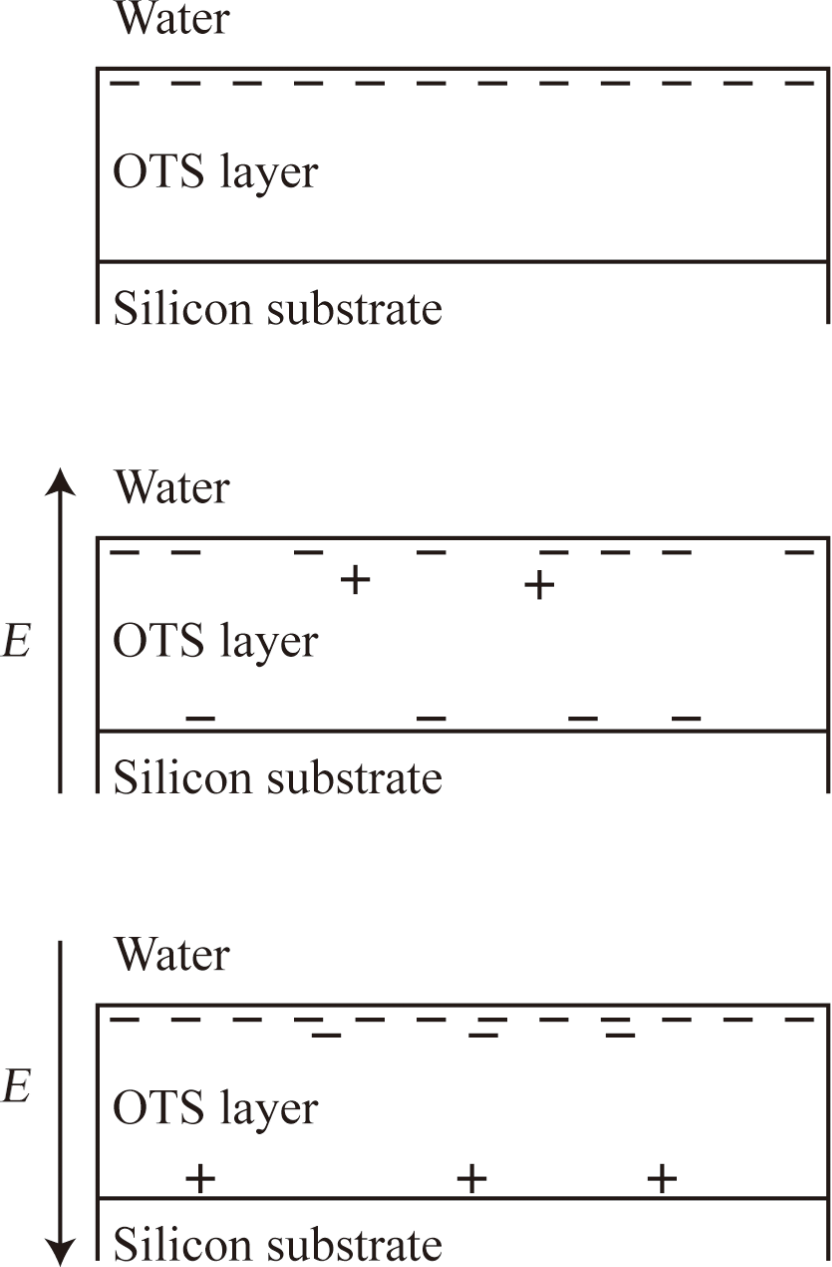
Schematic of the explanation for the results of electrostatic force measurement with positive voltage to the substrate, reproduced with permission from [94]. Copyright (2013) Elsevier.

As mentioned in section 2.2.2, the probe is negatively charged in DI water, then based on the repulsive electrostatic force measured between glass surface and OTS surface, the OTS surface can also be believed to be negatively charged, which is in agreement with the research by Audry et al. [93]. The change of surface charge density with applied positive and negative voltage can be simply explained by Figure 23. When a positive voltage is applied to the substrate, the OTS layer is placed in an electric field. Because of the existence of the electric field, electrons in the OTS layer are driven by the electric field moving from top to the bottom. As a result, the surface charge density decreases. When the applied voltage is negative, the direction of the electric field is opposite and the electrons in the OTS layer move from bottom to the top. However, because the top of OTS layer has already been negatively charged, there will be a large impact on the charge of the OTS surface. Thus, the electrostatic force with applied negative voltage to the substrate does not change [94].

**Figure 24:**
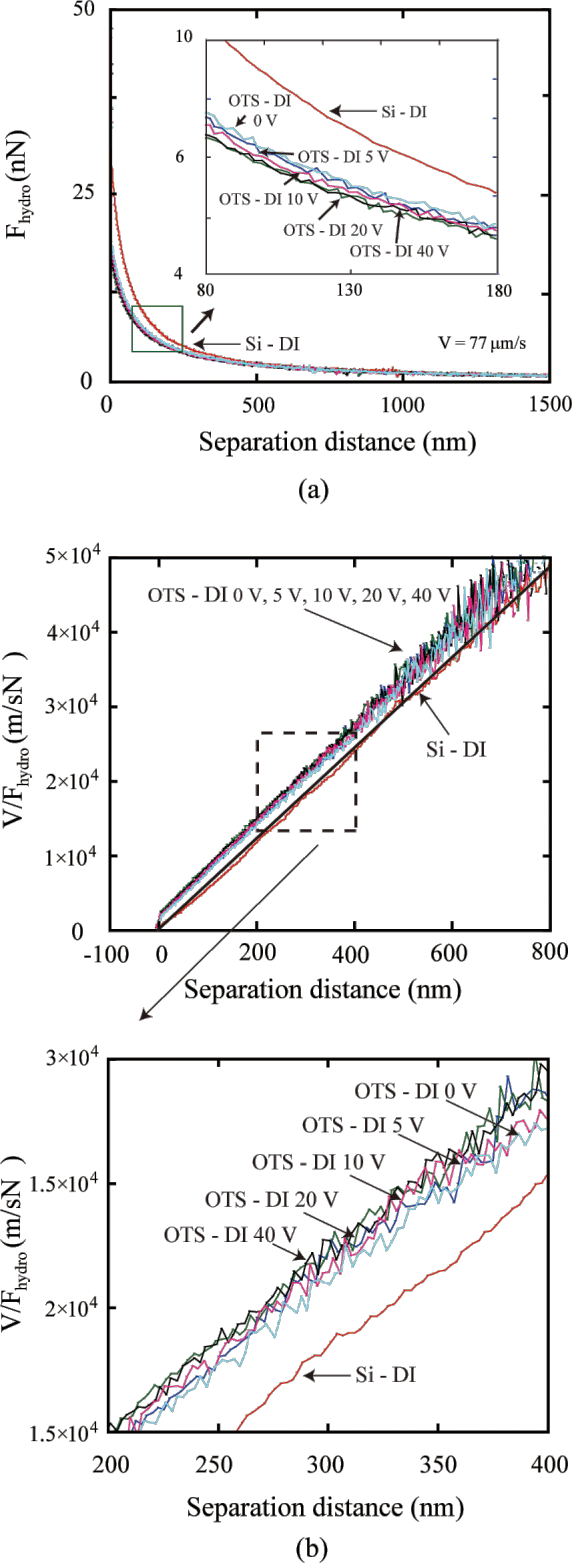
(a) Hydrodynamic force with high driving velocity (77 μm/s) on a silicon surface and an OTS surface in DI water with different positive voltages applied to the substrate, (b) *V/F*_hydro_ with high driving velocity (77 μm/s) on a silicon surface and an OTS surface in DI water with different positive voltages applied to the substrate. Reproduced with permission from [94]. Copyright (2013) Elsevier.

**Figure 25:**
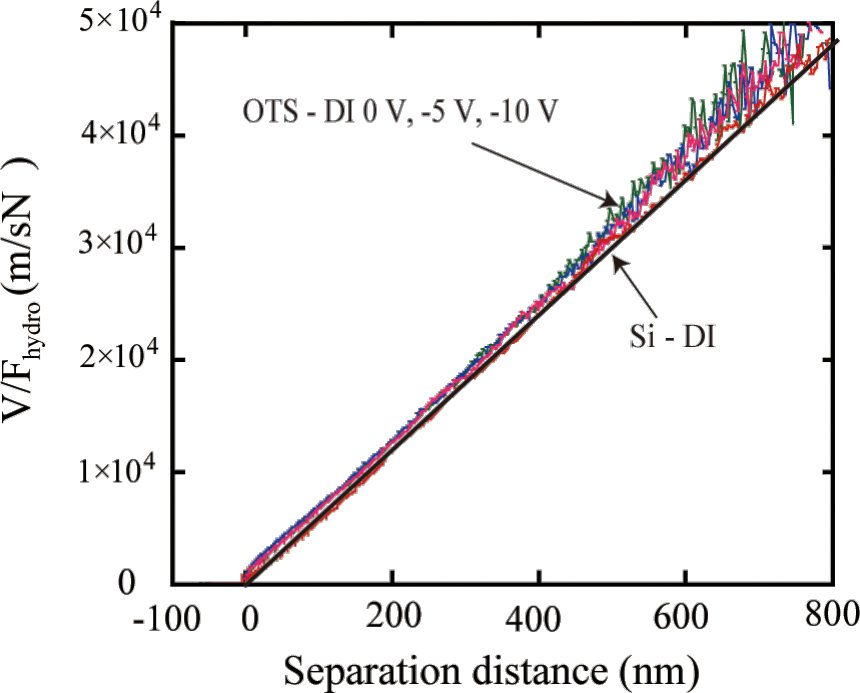
*V/F_hydro_* of the sphere with high driving velocity (77 μm/s) on a silicon surface and an OTS surface in DI water with different negative voltages applied to the substrate. Reproduced with permission from [94]. Copyright (2013) Elsevier.

**Figure 26:**
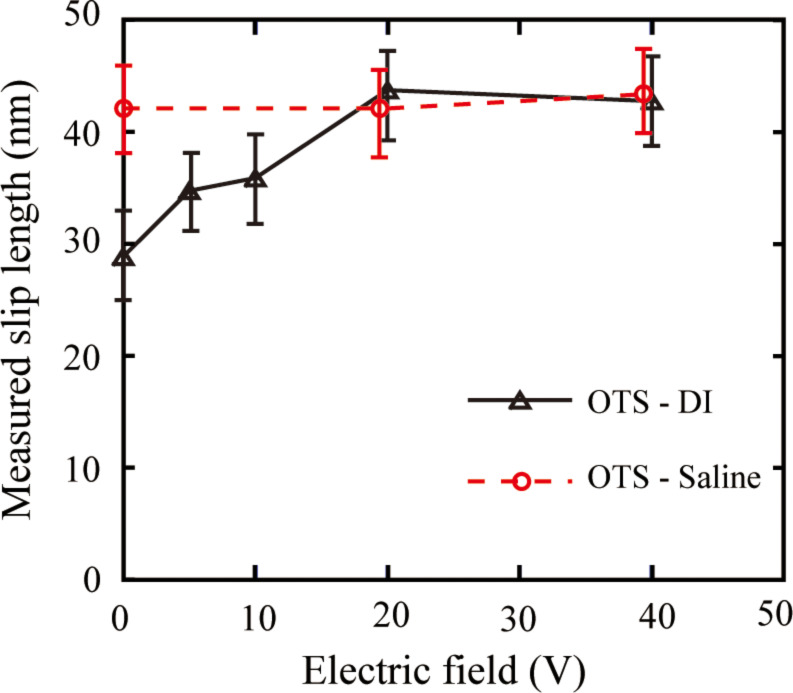
Measured slip lengths with different positive voltages applied to the substrate. Reproduced with permission from [94]. Copyright (2013) Elsevier.

To obtain the slip lengths with applied voltage, *V/F*_hydro_ at high driving velocities with positive and negative voltages applied to the substrate are shown in Figure 24 and Figure 25. The slip length can be obtained as the intercept of the plot at the separation distance. Thus the shift of the plot to the left with increasing applied positive voltage shows an increase of the slip length. The obtained slip lengths in DI water as a function of different positive voltages applied to the substrate are shown in Figure 26. With negative voltage to the substrate, the plots are almost superposed with each other, which means the slip length remained constant with different negative voltage to the substrate [94].

In DI water, the change of boundary slip can be understand as a result of the change of surface charge density. The slip length increased when the surface charge density decreased. The observation is in agreement with the previous discussion.

**Figure 27:**
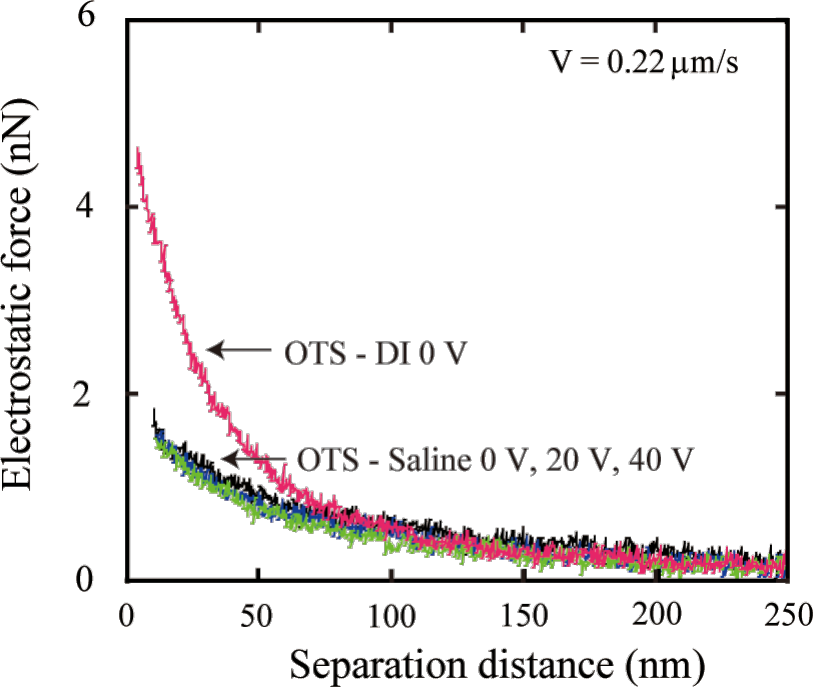
Electrostatic force in DI water and in saline with different positive voltages applied to the substrate. The voltage was varied from 0 to 40 V. (Sample was destroyed at higher voltage). Reproduced with permission from [94]. Copyright (2013) Elsevier.

**3.3.2 Saline:** Figure 27 shows the measured electrostatic force in saline with applied voltage. The data is presented as a function of the separation distance. The electrostatic force in DI water without any voltage is also shown as a reference. The electrostatic force in saline did not show an obvious change with an increasing applied voltage and was much smaller than that in DI water. The ion density in saline is larger than that in DI water, leading to a smaller Debye length, which in turn results in the smaller electrostatic force measured in saline. With applied positive voltage, the surface charge density may have a similar trend as that in DI water, but the change should be small. The equipment may not able to measure the small change of the small electrostatic force [94].

**Figure 28:**
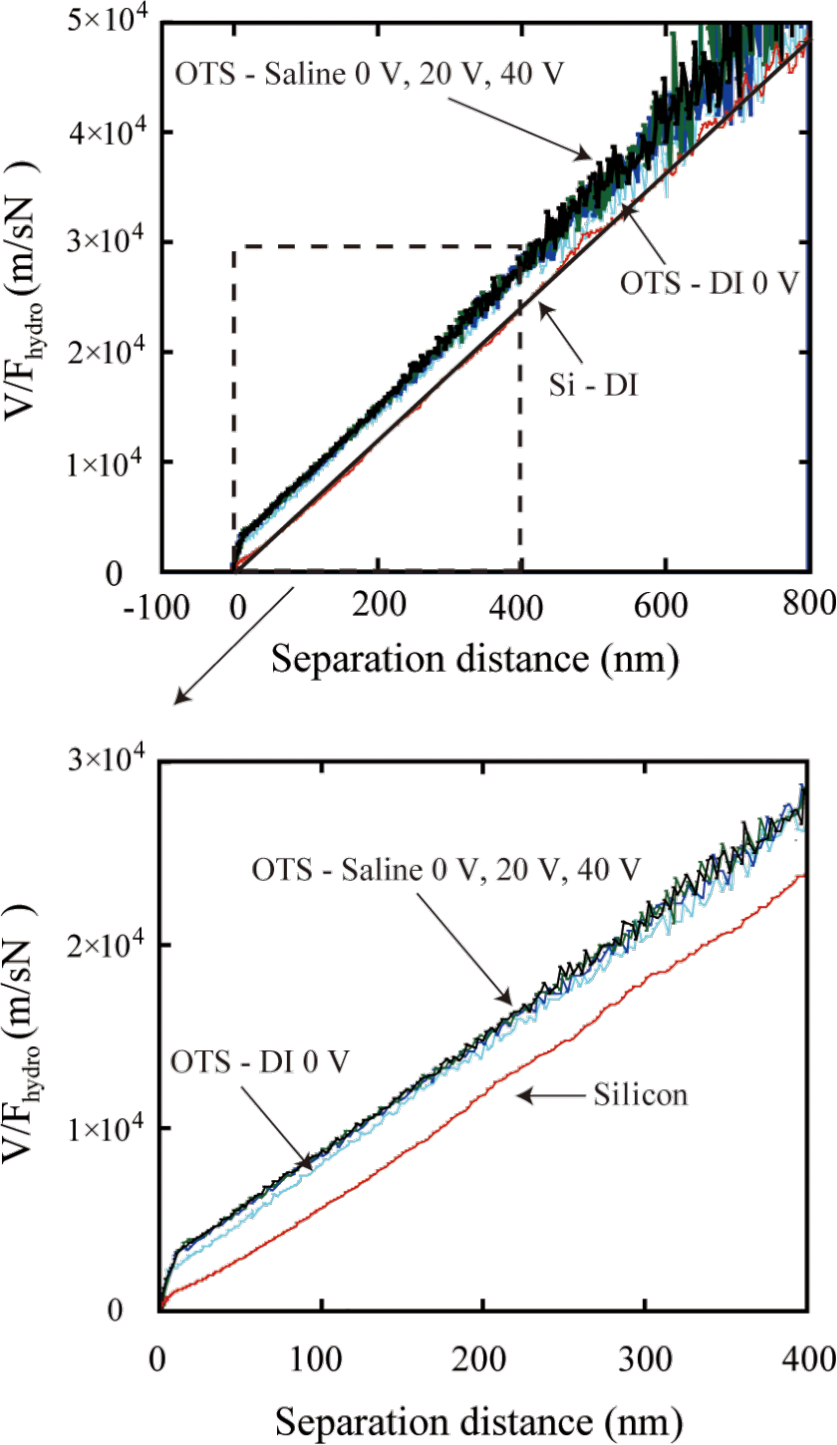
*V/F*_hydro_ plot of the sphere with high driving velocity (77 μm/s) on a silicon surface, an OTS surface in DI water and an OTS surface in saline with different positive voltages applied to the substrate. Reproduced with permission from [94]. Copyright (2013) Elsevier.

To obtain the slip length with applied voltage in saline, Figure 28 shows *V/F*_hydro_ with applied positive voltages as a function of the separation distance in saline with a high driving velocity of 77 μm/s. It is obvious that the measured slip length in saline was larger than that in DI water. With applied positive voltage, the plots are all almost superimposed, which means the slip length did not change significantly. The slip lengths in saline are also shown in Figure 26. The data are presented as a function of different applied positive voltages to the substrate. In saline, the electrostatic force is smaller than that in DI water. The measured slip length in saline is larger than that in DI water. With applied voltage, both the electrostatic force and the slip length did not show an obvious change. The observation in saline is also in agreement with the previous discussion.

#### 3.4 Summary

The effect of applied voltage on the drag of fluid flow is studied on a OTS surfaces in DI water and saline. The electrostatic force was measured to show the change of surface charge density. The slip length was measured as well. In DI water, the surface charge density decreased with an increasing positive voltage applied to the substrate. The drag of fluid flow is believed to be reduced by reducing the streaming potential. On the other hand, the slip length increased with applied positive voltage to the substrate, which provides a further reduction of the drag of fluid flow. With a negative voltage applied to the substrate in DI water, the surface charge density and the boundary slip did not show an obvious change. In saline, the measured electrostatic force is smaller than that in DI water while the measured slip length is larger than that in DI water. With applied voltage, the electrostatic force and the slip length did not show an obvious change. The observation in DI water and saline is in agreement with the discussion that the boundary slip can be affected by the surface charge density: A stronger surface charge will lead to smaller effective boundary slip.

Some studies show that a larger hydrophobicity will lead to larger boundary slip. In this experiment, with an application of applied voltage, the hydrophobicity is expected to decrease, which was shown on a PS surface. However, the slip length either increases (in DI water) or remains constant (in saline) on the OTS surface.

Reducing surface charge density, which was achieved in the studies presented here by applying a voltage on the OTS surface in DI water, will result in smaller drag because of an increase in the slip length and a decrease of the streaming potential.

## Conclusion

**Figure 29:**
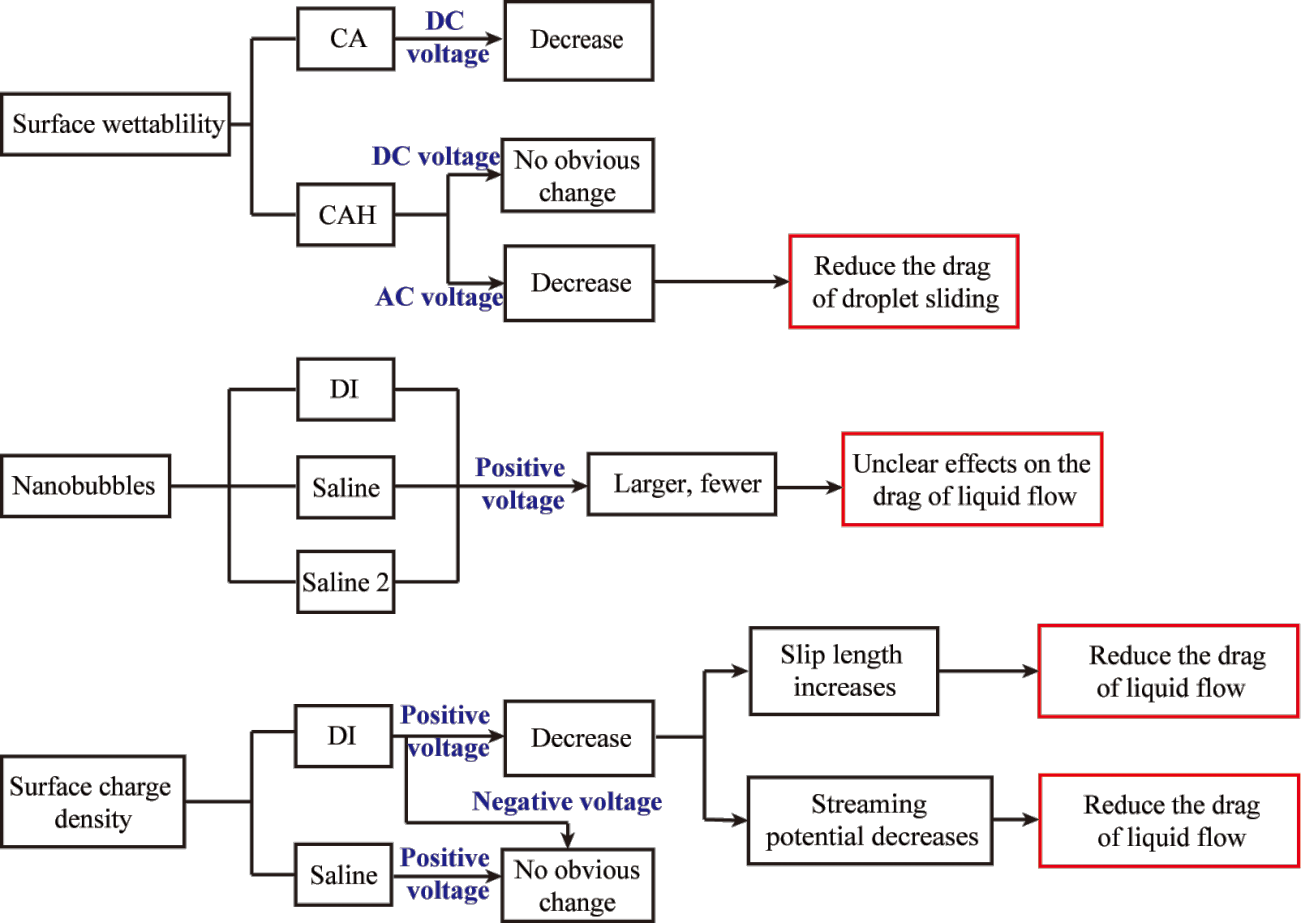
Summary of the experimental results for the influence of applied voltages on the surface wetting, nanobubbles, surface charge, boundary slip and the drag of liquid flow.

We have reviewed the recent research on the surface wetting, nanobubbles and boundary slip with an applied voltage. We tried to find the relationships between the surface charge density, slip length, nanobubbles, surface wetting and the drag of fluid flow based on the experiments by applying voltage. The experimental results are summarized in Figure 29. The surface wettability and nanobubbles were studied on PS surfaces and the surface charge density and boundary slip were studied on OTS surfaces, which have a larger CA and measureable slip length. The CAH on PS surface was found to decrease with an applied AC voltage. The nanobubbles on the PS surface in various liquids were found to be larger and fewer with a positive voltage applied to the substrate. The surface charge density in DI water on OTS surface was found to decrease with a positive voltage applied to the substrate. The slip length in DI water on an OTS surface was found to increase with a positive voltage applied to the substrate. By applying a voltage, the drag of fluid flow can be reduced in various aspects.

**Figure 30:**
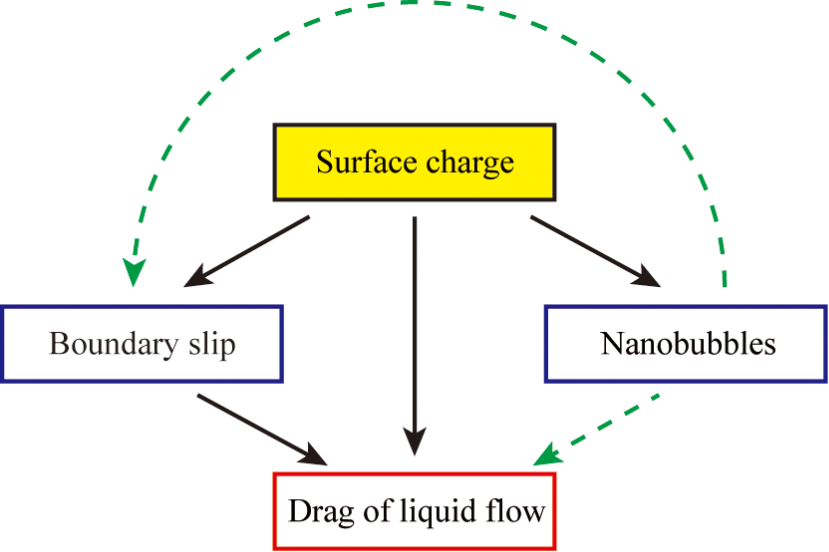
Schematic of the relationship between surface charge, boundary slip, nanobubbles and the drag of liquid flow.

When applying a positive voltage to the substrate, the surface charge density both on PS surfaces and on OTS surfaces decreases, which can explain the change of the nanobubbles on PS surfaces and the change of boundary slip on OTS surfaces. On OTS surfaces, the slip length in saline and saline 2 was measured to be constant at about 42 nm. The electrostatic force with applied voltage was also measured as a constant value in saline and saline 2, which can be an explanation for the same value of the measured slip length. The measured comparable values of slip length and surface charge together with the obviously different morphology of the nanobubbles in saline and saline 2 show that there may not be a direct effect of the nanobubbles on the boundary slip. Based on these studies, the relationship between surface charge, boundary slip, nanobubbles and the drag of liquid flow can be described as shown in Figure 30. The surface charge can affect the boundary slip, nanobubbles, and drag of liquid flow; the boundary slip can affect the liquid flow; the effect of nanobubbles on boundary slip and the drag of liquid flow are still unclear. Nanobubbles are believed to affect the drag of liquid flow and boundary slip, but more theoretical and experimental efforts are needed to be made to clarify the role of nanobubbles.

The surface charge density was studied qualitatively by comparing the electrostatic force on a probe in this review. To verify the influence of the surface charge density on the boundary slip, the surface charge density is needed to be studied quantitatively. The streaming potential was analyzed with a fixed value of slip length to simplify the simulation. When the surface charge density decreases, the increase in slip length will enhance the streaming potential. Furthermore, when applying a voltage, the zeta potential will be affected by the electric field. Thus, a more complicated model considering changes of boundary slip and the influence of the applied voltage on the zeta potential is needed.
